# Bolus ingestion of individual branched-chain amino acids alters plasma amino acid profiles in young healthy men

**DOI:** 10.1186/2193-1801-3-35

**Published:** 2014-01-17

**Authors:** Takuya Matsumoto, Koichi Nakamura, Hideki Matsumoto, Ryosei Sakai, Tomomi Kuwahara, Yoshihiro Kadota, Yasuyuki Kitaura, Juichi Sato, Yoshiharu Shimomura

**Affiliations:** Department of General Medicine/Family and Community Medicine, Nagoya University Graduate School of Medicine, Nagoya, 466-8560 Japan; Laboratory of Nutritional Biochemistry, Department of Applied Molecular Biosciences, Graduate School of Bioagricultural Sciences, Nagoya University, Nagoya, 464-8601 Japan; Institute for Innovation, Ajinomoto Co., Inc., Kawasaki, 210-8681 Japan

**Keywords:** Branched-chain amino acids, Plasma amino acids profile, Oral ingestion, Maximum concentration, Area under curve, Mood states

## Abstract

Physiological conditions in humans affect plasma amino acid profiles that might have potential for medical use. Because the branched-chain amino acids (BCAAs) leucine, isoleucine and valine are used as medicines and supplements, we investigated the acute effects of individual BCAAs (10–90 mg/kg body weight) or mixed BCAAs ingested as a bolus on plasma amino acid profiles in young healthy men. Plasma leucine levels rapidly increased and peaked around 30 min after leucine ingestion. Concentrations of plasma isoleucine, valine and phenylalanine subsequently decreased after ingestion, and those of methionine and tyrosine tended to decrease. The effects of ingested leucine on other plasma amino acids were biphasic, being higher at lower doses (10–20 mg/kg body weight). Isoleucine or valine intake also caused corresponding plasma amino acid concentrations to rapidly elevate, and peaks at 30–40 min after ingestion were much higher than that of plasma leucine after leucine ingestion. However, the increase in plasma isoleucine and valine concentrations essentially did not affect those of other plasma amino acids. The rate of decline among peak plasma BCAA concentrations was the highest for leucine, followed by isoleucine and valine. Oral mixed BCAAs promoted the decline in plasma isoleucine and valine concentrations. These results suggest that plasma leucine is a regulator of the plasma concentrations of BCAAs, methionine and aromatic amino acids.

**Electronic supplementary material:**

The online version of this article (doi:10.1186/2193-1801-3-35) contains supplementary material, which is available to authorized users.

## Introduction

Leucine, isoleucine and valine have branched-chain residues and are thus called branched-chain amino acids (BCAAs). All three are essential amino acids and account for about 35% of the indispensable amino acids in muscle proteins and about 40% of the preformed amino acids required by mammals (Harper et al. 1984). These amino acids uniquely share a common membrane transport system (Hyde et al. 2003) as well as the enzymes required for the first two steps of their catabolic systems (Harper et al. 1984).

Recent studies have demonstrated that BCAAs, particularly leucine, play an important role in the regulation of protein metabolism, because leucine stimulates protein synthesis by activating mammalian target of rapamycin (mTOR) complex 1 and suppressing protein degradation by inhibiting autophagy (Hands et al. 2009). Furthermore, accumulating evidence indicates that BCAAs play important roles in glucose and lipid metabolism (Nishitani et al. 2005; Doi et al. 2007; Arakawa et al. 2011; Kadota et al. 2012). Thus, BCAAs serve as medicines for treating liver cirrhosis (Kawaguchi et al. 2011) and as sport dietary supplements (Gleeson 2005). Supplementation with BCAAs normalizes amino acid profiles, ameliorates complications such as encephalopathy and hypoalbuminemia in patients with liver cirrhosis (Kawaguchi et al. 2011) and helps to reduce exercise-induced muscle damage and delayed-onset muscle soreness (Skillen et al. 2008; Shimomura et al. 2010).

Many studies have examined the influence of BCAAs on plasma amino acid profiles and protein metabolism in humans (Matthews 2005). Plasma amino-acid profiles have received considerable clinical focus because multivariate indexes consisting of plasma amino acid profiles have potential for diagnostic applications, disease activity monitoring and the assessment of pathophysiological conditions (Noguchi et al. 2006; Hisamatsu et al. 2012). The initial rationale for the clinical application of plasma amino acid profiles was Fischer’s ratio, which indicates the balance between BCAAs and aromatic amino acids (AAAs) and serves as a diagnostic marker and indicator of the progression of liver fibrosis and of the effects of drugs (Fischer et al. 1976; Soeters and Fischer 1976). These reports generated interest in BCAAs and many studies in humans have investigated the effects of varying amounts and durations of infused amino acids (Matthews 2005). Leucine infusion elevates blood leucine concentrations ~6-fold from the basal level and obviously decreases the concentrations of isoleucine (55%), valine (40%), methionine (50%), phenylalanine (35%) and tyrosine (35%) (Hagenfeldt et al. 1980). In contrast, isoleucine or valine infusion elevates corresponding amino acid concentrations to ~6.5- and ~11-fold, respectively, but do not significantly affect the concentrations of other amino acids (Eriksson et al. 1981). These findings indicated that leucine lowers AAA and methionine concentrations more effectively than other BCAAs.

On the other hand, less is known about the effects of various oral doses of individual BCAAs than of essential amino acid mixtures upon humans. The profiles of plasma amino acids after ingesting amino acid supplements should be clarified because the availability of circulating amino acids regulates muscle protein turnover (Wolfe 2002; Bohé et al. 2003; Drummond et al. 2010). Exercise and essential amino acid supplements, especially those providing added leucine, stimulate muscle protein synthesis (Pasiakos and McClung 2011) and the plasma profiles of amino acid concentrations after amino acid ingestion are affected during strength training (Mero et al. 2008; 2009). Since BCAAs are commonly included among supplements in sports drinks, the effects of ingesting various doses of leucine, isoleucine and valine on plasma amino acid profiles in humans require clarification. Thus, the present study investigated the effects of ingesting 10–90 mg/kg body weight (BW) of individual BCAAs and a mixture of BCAAs on plasma amino acid profiles in young healthy men.

## Materials and methods

### Reagents

Individual BCAAs were provided by Ajinomoto Co. Inc. (Tokyo, Japan). Heparin was purchased from Mochida Pharmaceutical Co. Ltd. (Tokyo, Japan). All other reagents were of biochemical grade and were obtained from Wako Pure Chemical Industries Ltd. (Osaka, Japan).

### Experiments

Several series of investigations into the ingestion of individual BCAAs or mixed BCAAs proceeded between June 2009 and September 2011. Five healthy young males ingested 10, 20, 30, 45, 60, 75 and 90 mg/kg BW (seven doses of individual BCAAs; 21 experiments) and 63 and 94.5 mg/kg BW of mixed BCAAs (two experiments) and a control experiment was conducted without amino acids. These 24 experiments proceeded in the order of control (no amino acids), followed by the oral ingestion of leucine, isoleucine, BCAA mixtures and valine. Low to high doses of amino acids were investigated in the series of experiments with individual BCAAs or BCAA mixtures. All experiments were conducted at intervals of at least 1 week.

### Participants

We recruited five healthy male undergraduate or graduate students at Nagoya University. After completing the initial control and leucine ingestion experiments, only one participant was replaced. The means ± SEM for age, height and body weight of the participants for the initial control and leucine ingestion experiments were 22.4 ± 0.9 years, 175 ± 2 cm and 66.6 ± 2.1 kg, respectively, and after replacing the single participant, these values were 22.6 ± 0.6 years, 177 ± 2 cm and 67.2 ± 0.8 kg, respectively. The absence of liver failure, diabetes or dyslipidemia was determined in the participants from interviews and blood tests of glucose, insulin, triglyceride, total cholesterol, alanine aminotransferase (ALT) and aspartate aminotransferase (AST) levels before starting the study.

The study design, purpose and possible risks were explained to the students, who then provided written inform consent to participate in the present study. The Human Research Review Committee of the Nagoya University School of Medicine (Nagoya, Japan) approved the study protocol.

### Test drinks

Each BCAA was given to the participants in solution (partly as a suspension) in 200 ml/60 kg BW of distilled water at concentrations of 0.3%, 0.6%, 0.9%, 1.35%, 1.8%, 2.25% and 2.7% (w/v) for the respective doses of 10, 20, 30, 45, 60, 75 and 90 mg amino acids/kg BW. Leucine did not fully dissolve at concentrations ≥1.8%. Test solutions of 1.89% and 2.84% (w/v) BCAA mixtures (weight ratio of BCAAs (Ile:Leu:Val), 1:2:1.2; Kawaguchi et al. 2011) for the respective doses of 63 and 94.5 mg BCAAs/kg BW were prepared in distilled water. These corresponded to leucine doses of 30 and 45 mg/kg in a volume of 200 mL/60 kg. The control was 200 mL of distilled water/60 kg.

### Experimental design

The participants refrained from vigorous physical activity or ingesting alcohol or any other fluids and consumed the same meal (Japanese-style boxed meal) for dinner at 18:00–20:00 on the day before experiments. Thereafter, the participants were allowed to ingest only water until the experiment started.

On the day of experiments, participants fasted overnight and ate one ball of rice (about 160 kcal, 90% carbohydrate at the laboratory at 08:30) to reduce the effects of starvation on BCAA catabolism. Blood samples (~6 mL at 0 and 240 min; ~4 mL for time points in between) collected about 1 h later and then at 15, 30, 45, 60, 75, 90, 105, 120, 180 and 240 min after consuming a test drink containing 0–90 mg amino acid/kg BW were immediately heparinized, cooled in ice, and then plasma was separated by centrifugation at 4°C. Free amino acids were analyzed in plasma samples that were deproteinized with 5% (w/v) trichloroacetic acid (Noguchi et al. 2006). Deproteinized samples were stored at -80°C.

The effects of amino acid ingestion on mood states were analyzed using the self-reporting, brief Japanese version of the Profile of Mood States (POMS) (Yokoyama et al. 1990). This survey is based on a 30-item mood checklist designed to provide information on six mood states. The participants evaluated the degree of each mood before blood was collected and at 120 min after consuming the test drink during all experiments using a rating scale from 0 (not at all) to 4 (extremely). Scores for each separate mood state were calculated by summing the responses to all items in each of the following subscales: tension-anxiety, depression-dejection, anger-hostility, confusion, fatigue and vigor.

### Analyses

Free amino acid concentrations were measured using an automated amino acid analyzer (L-8800: Hitachi, Tokyo, Japan). Concentrations of plasma glucose (Banauch 1975), insulin (Morgan and Lazarow 1962), free fatty acids (FFA) (Sugo et al. 1990), total cholesterol (Richmond 1973), LDL-cholesterol (Friedewalt 1972) and urea (Morishita et al. 1997) were measured as described at all time-points and ALT and AST activities (Miura 1995) were measured only at 0 and 240 min. All plasma components except amino acids were measured at Special Reference Laboratories Inc. (Tokyo, Japan).

### Statistics

Data are expressed as means ± SEM unless otherwise stated. Changes in the concentrations of plasma components over time within each BCAA dose were analyzed using a one-way repeated measures (rm) ANOVA. The effects of BCAA intake on the profiles of plasma amino acids and other components over time were determined using a two-way rmANOVA (11 × 8 doses of individual BCAAs, and 11 × 2 doses of mixed BCAAs). Significant effects of BCAA ingestion were further analyzed using Tukey’s post-hoc test to determine significant differences between doses. Correlations between amino acid doses and the maximum (or minimum) concentrations (Cmax or Cmin), and between amino acid doses and the area under the curves (AUC) of amino acid plasma concentrations were analyzed as described (Motulsky and Christopoulos 2004). Biphasic correlations between the two parameters were analyzed using a standard, continuous two-phase linear regression model. Briefly, the slopes and y-intercepts of two-segmented lines were optimized by minimizing the sum of the squares of the vertical distances of the points from the lines using the Marquardt method, and the breakpoint was defined as their crossing point. The likelihood of the method was analyzed by comparing the fit with that in a simple linear regression model using Akaike’s information criteria. Differences in POMS scores before and after consuming test drinks were analyzed using the Wilcoxon signed-rank test. Differences were considered statistically significant at *P* < 0.05.

## Results

### Effects of oral leucine on plasma amino acid profiles and concentrations of other plasma components

#### Repeated-measures ANOVA

The effects of various doses of oral leucine on plasma concentrations of free amino acids over time were statistically analyzed using two-way (8 doses × 11 time points) rmANOVA. The profiles of the three BCAAs and phenylalanine were significantly affected (*P* < 0.001 for all three BCAAs; *P* = 0.005 for phenylalanine), and that of plasma methionine tended to be affected (*P* = 0.071) by oral leucine.

Changes in the concentrations of plasma amino acids over time within each dose of leucine were also analyzed. The results of one-way rmANOVA showed that all doses of leucine significantly affected the concentrations of methionine, phenylalanine and tyrosine over time (Additional file 1: Table S1), and that higher doses significantly affected those of tryptophan (Additional file 1: Table S1) and histidine (Additional file 1: Table S2). Oral leucine did not affect the concentrations of any other amino acids (Additional file 1: Table S2).

#### Profiles of plasma BCAA concentrations

**Figure 1:**
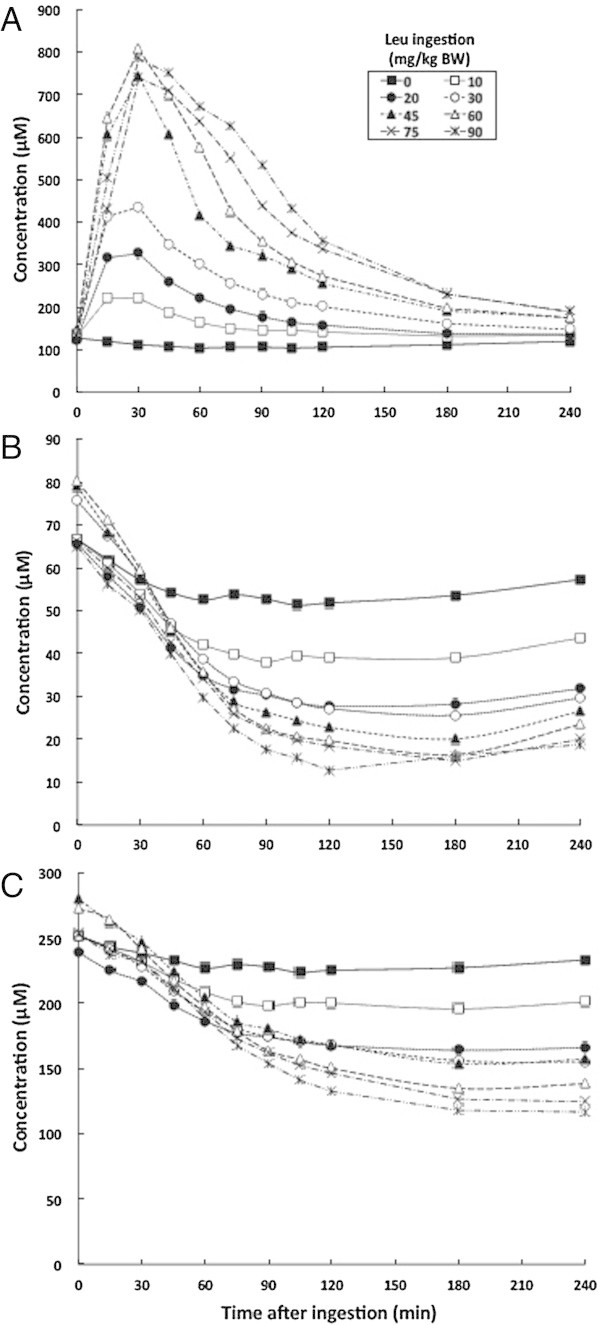
**Mean changes in plasma BCAA concentrations at various time points after leucine ingestion. A**, leucine; **B**, isoleucine; **C**, valine.

Plasma leucine concentrations remained unchanged during the control experiment, but rapidly increased after ingesting various doses of leucine (Additional file 1: Table S1 and Figure 1A). The plasma concentration peaked at 15–30 min and at around 30 min after ingesting 10–30 and 45–90 mg/kg BW, respectively. The peak increased at up to 45 mg leucine/kg BW. Plasma leucine concentrations gradually decreased after reaching a peak at all doses, but the decline was slower at increasing doses between 45 and 90 mg/kg BW. Plasma leucine concentrations remained close to control levels at 240 min, but were still significantly higher at doses >45 mg/kg BW compared with controls (176 ± 16 vs.120 ± 2 μM).

Leucine ingestion gradually decreased plasma isoleucine (Additional file 1: Table S1 and Figure 1B) and valine (Additional file 1: Table S1 and Figure 1C) concentrations, which reached a nadir 90–120 min later and were sustained until 240 min in all experiments. Higher doses of leucine caused more obvious effects. The levels of plasma isoleucine and valine were the lowest at 120–240 min after ingesting 90 mg/kg BW of leucine, being 25% and 50% of respective control values at the same time point.

#### Cmax or Cmin, and AUC of plasma amino acids

**Figure 2:**
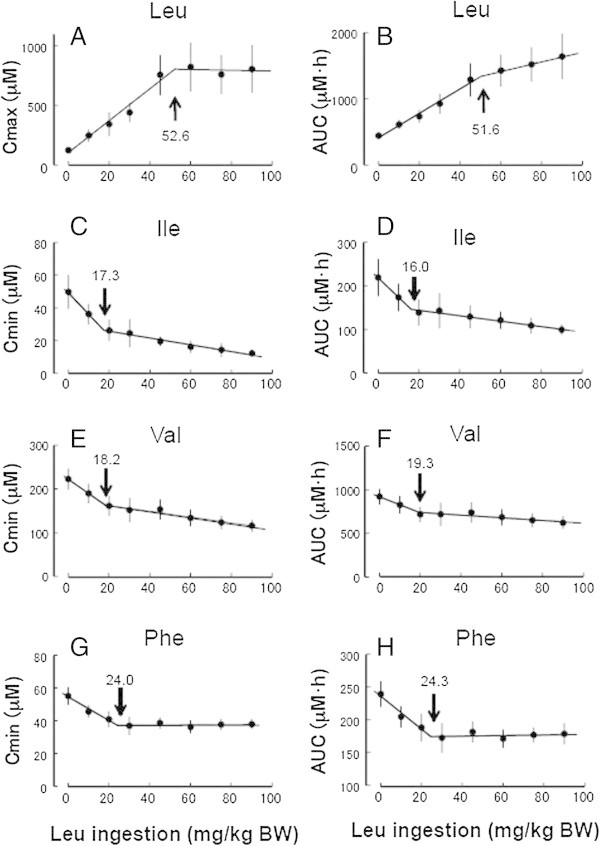
**Correlations between ingested leucine and Cmax, Cmin and AUC of plasma amino acid concentrations after leucine ingestion.** Cmax and AUC of plasma leucine **(A, B)**, and Cmin and AUC pf plasma isoleucine **(C, D)**, valine **(E, F)** and phenylalanine **(G, H)** concentrations. Arrows, breakpoints.

The Cmax of plasma leucine was increased by increasing the dose of leucine up to 45 mg/kg BW and it reached a plateau at 53 mg/kg BW (Figure 2A). Linear regression determined from the AUC for plasma leucine concentrations was biphasic in terms of leucine dose (Figure 2B). The slope of the regression line was >2-fold higher in the initial phase for the lower doses (0–45 mg/kg BW; Slope = 18.4) than in the later phase for higher doses (60–90 mg/kg BW, Slope = 7.1), and the breakpoint of the two-segmented lines was about 52 mg/kg BW. This breakpoint was almost the same as the dose of leucine at which the Cmax reached a plateau.

Linear regression of Cmin and the AUC (Figure 2C and D, respectively) of the plasma isoleucine concentration was biphasic in terms of leucine dose. The rate at which the plasma isoleucine concentration decreased was much greater at the lowest dose (10 mg/kg BW) than 20–90 mg/kg BW, indicating that the lower dose of leucine was sufficient to decrease the plasma isoleucine concentration. The breakpoints of the two-segmented line were 17 and 16 mg leucine/kg BW for the Cmin and the AUC, respectively. These breakpoint values are much lower than those for the Cmax and AUC of plasma leucine.

The trends for Cmin and the AUC (Figure 2E and F, respectively) of the plasma valine concentration were the same as those for plasma isoleucine, and the breakpoint of the two-segmented line was 18 and 19 mg leucine/kg BW, respectively.

The profile of changes plasma phenylalanine induced by oral leucine were similar to that of plasma valine (Additional file 1: Table S1), but the Cmin (Figure 2G) and AUC (Figure 2H) of the plasma phenylalanine concentration remained essentially constant at doses of 30–90 mg/kg BW. The lowest level of plasma phenylalanine was about 67% of controls. The breakpoint of the two-segmented line was 24 mg leucine/kg BW for both Cmin and AUC.

#### Concentrations of other plasma components

The concentrations of plasma components other than amino acids over time were not significantly affected by leucine (Additional file 2: Online Resource 1). Although changes in plasma glucose, insulin and FFA concentrations over time within each leucine dose were significant in the analyses with the one-way rmANOVA, changes were also similarly time-dependent after vehicle ingestion.

### Effects of isoleucine ingestion on plasma amino acid profiles and concentrations of other plasma components

#### Repeated-measures ANOVA

The results of statistical analyses using two-way (8 doses × 11 time points) rmANOVA showed that oral isoleucine significantly (*P* < 0.001) affected only the plasma isoleucine profile. One-way rmANOVA showed that all doses of isoleucine significantly affected the plasma concentrations of leucine, isoleucine and methionine over time, although the changes in plasma leucine and methionine concentrations were quite minimal (Additional file 1: Table S3). The concentrations of other amino acids were essentially unaffected by oral isoleucine (Additional file 1: Table S4).

#### Profiles of plasma BCAA concentrations

**Figure 3:**
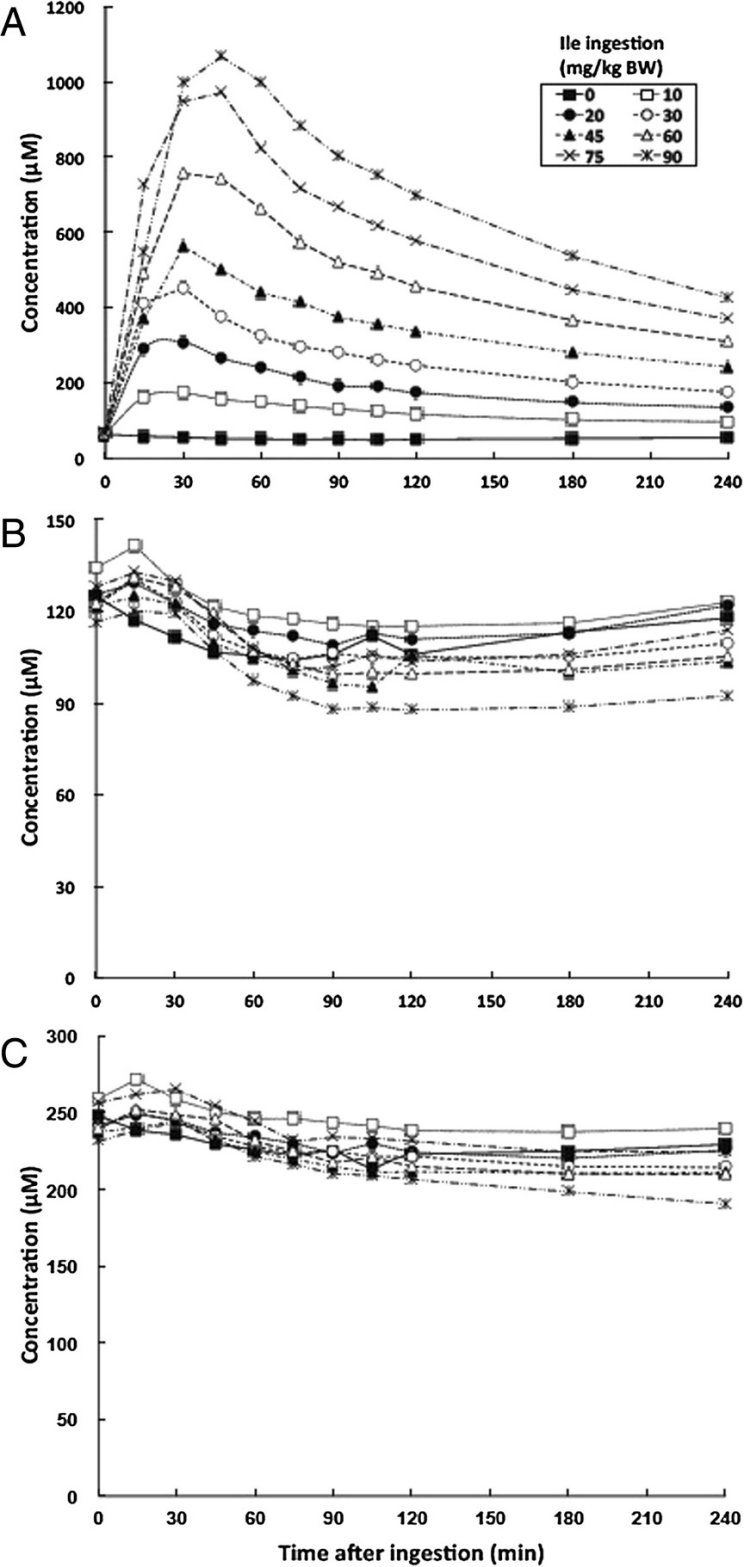
**Mean changes in plasma BCAA concentrations at various time points after isoleucine ingestion. A**, isoleucine; **B**, leucine; **C**, valine.

Various doses of oral isoleucine increased plasma isoleucine concentrations (Additional file 1: Table S4 and Figure 3A). The plasma concentration of isoleucine peaked at 30 min after ingesting 10–60 mg/kg BW of isoleucine, similarly to that of plasma leucine concentrations after ingesting leucine, and at 45 min after ingesting 75–90 mg/kg BW. The plasma isoleucine peaks were elevated with increasing doses of isoleucine up to 90 mg/kg BW. Plasma isoleucine concentrations gradually declined after reaching a peak at all ingested doses, but more slowly than the plasma leucine concentration after leucine ingestion; plasma isoleucine and leucine concentrations were >6-fold and 1.4-fold, respectively, of the base at 240 min after the highest dose (90 mg/kg BW) of corresponding amino acid ingestion. All tested doses of isoleucine had apparently minor effects on plasma leucine (Additional file 1: Table S3, Figure 3B) and valine (Additional file 1: Table S3, Figure 3C) concentrations over time.

These results indicate that plasma amino acid profiles were much less affected by oral isoleucine than by oral leucine.

#### Cmax and AUC of the plasma isoleucine concentrations

**Figure 4:**
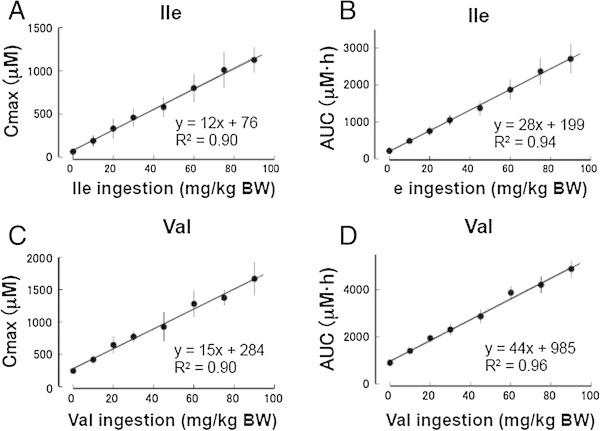
**Correlations between ingested isoleucine and valine with Cmax or AUC of respective plasma amino acid concentration.** Cmax and AUC of plasma isoleucine **(A, B)** and valine **(C, D)** concentrations after ingesting isoleucine and valine, respectively.

In contrast to leucine, the Cmax (Figure 4A) and AUC (Figure 4B) of the plasma isoleucine concentrations linearly correlated with doses of isoleucine without a breakpoint.

#### Concentrations of other plasma components

Various doses of oral isoleucine, like leucine, did not affect the concentrations of other plasma components over time (Additional file 2: Online Resource 2).

### Effects of valine ingestion on plasma amino acid profile and concentrations of other plasma components

#### Repeated-measures ANOVA

Statistical analyses using two-way (8 doses × 11 time points) rmANOVA showed that valine ingestion significantly affected only the profile of plasma valine (*P* < 0.001). One-way rmANOVA showed that changes in the concentrations of plasma amino acids, except for valine, over time within each dose of valine were essentially not significant (Additional file 1: Table S5 and S6).

#### Profiles of plasma BCAA concentrations

**Figure 5:**
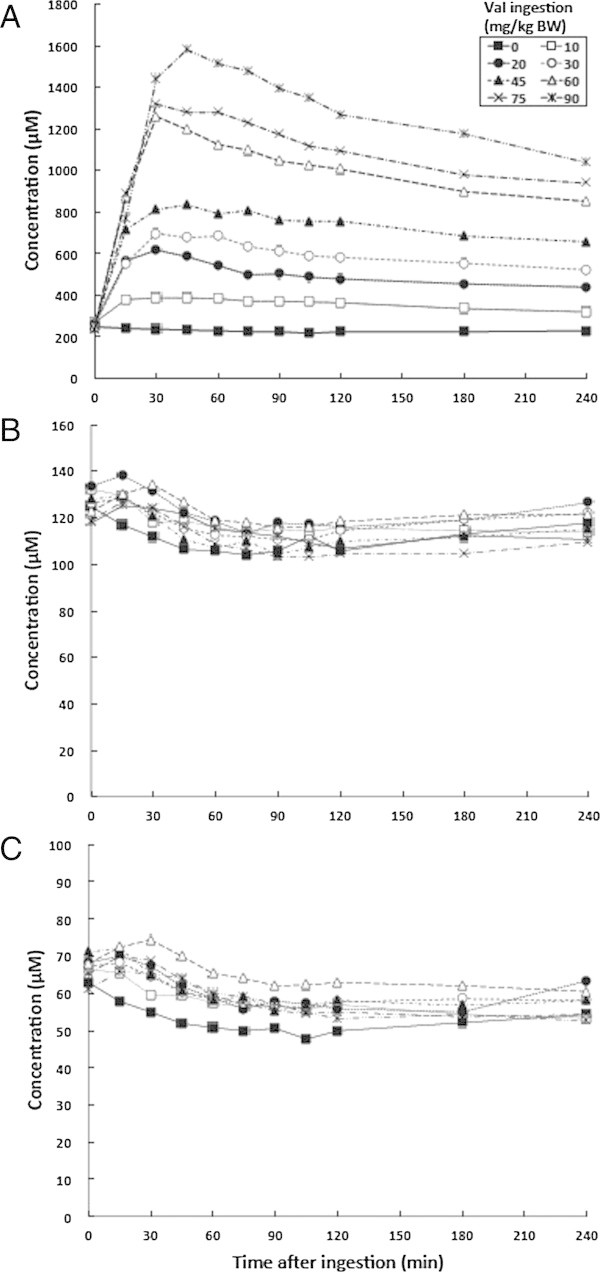
**Mean changes in plasma BCAA concentrations at various time points after valine ingestion. A**, valine; **B**, leucine; **C**, isoleucine.

Various doses of oral valine increased the concentrations of plasma valine to peak at 30–45 min after ingesting 10–90 mg/kg BW (Additional file 1: Table S5, Figure 5A). However, peaks for plasma valine after valine ingestion were much higher than those for plasma isoleucine after isoleucine ingestion at the same doses, reaching 1035 ± 28 μM at 240 min at the highest ingested dose of valine. Peak plasma valine concentrations then declined more slowly than those of leucine and isoleucine. The various doses of valine apparently had minimal effects on plasma leucine and isoleucine concentrations over time (Figure 5B and C respectively, and Additional file 1: Table S5).

These results indicate that valine induced the least amount of changes in plasma amino acid profiles among the three BCAAs.

#### Cmax and AUC of plasma valine concentrations

The Cmax and AUC (Figure 4C and D, respectively) of the plasma valine concentration linearly correlated with valine dose, similarly to that of the plasma isoleucine concentration after isoleucine ingestion.

#### Concentrations of other plasma components

The tested doses of valine, like leucine and isoleucine, did not alter the concentrations of plasma components other than amino acids over time (Additional file 2: Online Resource 3).

### Effects of mixed BCAAs on plasma amino acid profiles and concentrations of other plasma components

#### Repeated-measures ANOVA and profiles of plasma BCAA concentrations

We examined the effects of 63 and 94.5 mg/kg BW of mixed BCAA (including 30 and 45 mg/kg BW of leucine, respectively) on plasma amino acid profiles. Statistical analyses using two-way (3 doses × 11 time points) rmANOVA showed that mixed BCAA doses significantly (*P* < 0.001) affected the profiles of plasma leucine, isoleucine, valine, phenylalanine and tyrosine. Plasma BCAA levels peaked at 30–45 min (leucine and isoleucine) and at 45–60 min (valine) after ingesting the mixed BCAAs (Additional file 1: Table S7). Thus, the levels peaked somewhat later compared with individual BCAA ingestion. Plasma concentrations of isoleucine and valine after the peak (Additional file 1: Table S7) declined more rapidly than after ingesting either alone (Additional file 1: Table S3 and S5, respectively), suggesting that the increased plasma concentrations of leucine promoted the decline of isoleucine and valine concentrations that were increased by ingesting mixed BCAAs. These results are supported by the findings that the decline in peak plasma isoleucine and valine concentrations after ingesting the respective BCAA was much slower than that of plasma leucine after ingesting leucine. The concentrations of other amino acids were essentially unaffected by oral mixed BCAAs (Additional file 1: Tables S7 and S8).

#### Concentrations of other plasma components

The tested doses of mixed BCAAs, like individual BCAAs, did not change the concentrations of plasma components other than amino acids over time (Additional file 2: Online Resource 4).

### POMS scores

Table 1 shows that the POMS scores obtained before and at 2 h after ingesting maximal doses of individual BCAAs and the BCAA mixtures did not significantly differ, indicating that mood states were not significantly affected. In addition, none of the participants indicated discomfort or physical symptoms throughout the study, in accordance with the POMS scores.

**Table 1:** **POMS scores in the maximal doses of individual BCAAs and mixed BCAA ingestion**

Amino acid ingested	Mood	Before ingestion	2 h after ingestion	***P*** value
Basal	Tension - anxiety	0.2 ± 0.1	1.4 ± 0.6	0.66
	Depression - dejection	0.8 ± 0.4	0.6 ± 0.3	0.66
	Anger - hostility	0.8 ± 0.4	0.8 ± 0.4	1.00
	Confusion	2.6 ± 1.2	2.0 ± 0.9	0.59
	Fatigue	3.6 ± 1.6	3.4 ± 1.5	0.79
	Vigor	6.2 ± 2.8	2.6 ± 1.2	0.07
Leu 90 mg/kg BE	Tension - anxiety	0.8 ± 0.4	0.2 ± 0.1	0.32
	Depression - dejection	0.4 ± 0.2	0.2 ± 0.1	0.32
	Anger - hostility	0.0 ± 0.0	0.0 ± 0.0	1.00
	Confusion	1.6 ± 0.7	0.6 ± 0.3	0.32
	Fatigue	3.6 ± 1.6	3.0 ± 1.3	0.18
	Vigor	6.2 ± 2.8	6.6 ± 3.0	0.59
Ile 90 mg/kg BW	Tension - anxiety	0.0 ± 0.0	0.0 ± 0.0	1.00
	Depression - dejection	0.0 ± 0.0	0.0 ± 0.0	1.00
	Anger - hostility	0.4 ± 0.2	0.0 ± 0.0	0.32
	Confusion	0.2 ± 0.1	0.0 ± 0.0	0.32
	Fatigue	2.4 ± 1.1	2.0 ± 0.9	0.16
	Vigor	8.4 ± 3.8	9.0 ± 4.0	0.41
Val 90 mg/kg BW	Tension - anxiety	0.0 ± 0.0	0.0 ± 0.0	1.00
	Depression - dejection	0.0 ± 0.0	0.0 ± 0.0	1.00
	Anger - hostility	0.0 ± 0.0	0.2 ± 0.1	0.32
	Confusion	0.6 ± 0.3	0.2 ± 0.1	0.32
	Fatigue	2.0 ± 0.9	1.8 ± 0.8	0.32
	Vigor	10.6 ± 4.7	10.4 ± 4.7	0.32
Mixed BCAA 94.5 mg/kg BW	Tension - anxiety	0.0 ± 0.0	0.0 ± 0.0	1.00
	Depression - dejection	0.0 ± 0.0	0.0 ± 0.0	1.00
	Anger - hostility	0.0 ± 0.0	0.0 ± 0.0	1.00
	Confusion	0.0 ± 0.0	0.2 ± 0.1	0.32
	Fatigue	1.6 ± 0.7	1.4 ± 0.6	0.32
	Vigor	11.0 ± 4.9	11.2 ± 5.0	1.00

Values are means ± SE.

Statistic analyses were performed by the Wilcoxon signed-rank test.

## Discussion

The present study found that a bolus ingestion of 10–90 mg/kg BW of leucine significantly decreased plasma concentrations of isoleucine, valine and phenylalanine and tended to decrease those of methionine and tyrosine. Furthermore, the bolus ingestion of isoleucine or valine did not cause such effects. These findings are largely consistent with those of studies of individual BCAA infusions (Hagenfeldt et al. 1980; Eriksson et al. 1981) and of mixed BCAA ingestion (Shimomura et al. 2009; Zhang et al. 2011). However, we discovered that leucine at doses as low as 10–20 mg/kg BW can effectively decrease plasma levels of isoleucine, valine and phenylalanine. Furthermore, the rate at which the peak plasma BCAA concentrations declined obviously differed between with the presence and absence of leucine ingestion, suggesting that plasma leucine is a potent regulator of the plasma concentrations of BCAAs, methionine and AAAs.

Individual BCAA intake increased corresponding plasma BCAA concentrations, which peaked around 30 min later. However, the heights of the peaks in response to the tested doses of individual BCAAs differed between leucine and other BCAAs. Plasma leucine levels after leucine ingestion reached a plateau (at about 800 μM) after ingesting at 53 mg leucine/kg BW, whereas plasma isoleucine and valine levels after ingestion of each respective amino acid dose-dependently increased up to 90 mg/kg BW. These results suggest that the absorption rate of leucine from the gut into the circulation reached a plateau at a dose of 53 mg/kg BW, although the capacity for leucine in human plasma appears to be much higher (about 2000 μM), as determined by a study that identified the tolerable upper intake level of leucine in young men (Elango et al. 2012). This phenomenon might be associated with the solubility of leucine, which did not completely dissolve at doses of >60 mg/kg BW in test drinks. This property of leucine also reflects the biphasic linear regression of the increase in the rate of the AUC of the plasma leucine concentration in response to dose with a breakpoint at about 52 mg/kg BW.

The increasing plasma leucine concentration peaked at around 30 min after ingestion and then plasma concentrations of other BCAAs decreased. The ingestion of 90 mg leucine/kg BW caused plasma isoleucine and valine concentrations to fall to around 25% and 50% of respective control values and remain at these levels for at least 4 h. All three BCAAs share the first two enzymes in their catabolic systems and branched-chain α-keto derived from leucine, but not from isoleucine or valine, promotes BCAA catabolism by activating the second enzyme (branched-chain α-keto acid dehydrogenase (BCKDH) complex) in the catabolic system of rat muscle and liver via inhibition of BCKDH kinase, which is responsible for inactivation of the complex by phosphorylation (Paxton and Harris 1984; Hood and Terjung 1987; Fujii et al. 1994; Ishiguro et al. 2006). Thus, promoting BCAA degradation might have contributed to the decreases in plasma isoleucine and valine levels. On the other hand, the consumption of free amino acids required for protein synthesis after ingesting leucine in amounts up to ≤90 mg/kg BW appeared to have essentially no effect on plasma amino acid profiles, like the concentrations of other essential amino acids such as lysine and threonine. This result is consistent with the fact that the leucine specifically induces a reduction in protein breakdown without increasing protein synthesis in humans (Matthews 2005), although leucine administration stimulates protein synthesis in the tissues of other animals (Proud 2007).

On the other hand, human muscle protein synthesis is enhanced by exercise (especially resistance exercise) during the post-exercise recovery period, and protein synthesis is greatly stimulated by resistance exercise combined with essential amino acid ingestion (Walker et al. 2011). The timing of essential amino acid supplementation in relation to exercise is important to stimulate human skeletal muscle protein synthesis; ingestion of a solution of essential amino acids and carbohydrate after, but not before, resistance exercise maximally stimulates muscle protein synthesis during recovery after exercise (Drummond et al. 2009; Fujita et al. 2009). Leucine is a key amino acid in the anabolic response of skeletal muscle to essential amino acids (Drummond et al. 2009; Pasiakos and McClung 2011).

Branched-chain amino acids are used as medicines to treat liver cirrhosis (Kawaguchi et al. 2011). The BCAA:AAA ratio plays a causative role (Bianchi et al. 2005; Dejong et al. 2007) in hepatic encephalopathy, and providing BCAA supplements to patients with liver cirrhosis ameliorates hepatic encephalopathy (Holecek 2010). This effect of BCAA supplementation might, at least in part, be explained by the leucine-induced decrease in plasma concentrations of AAAs, which might be incorporated into body tissues from the circulation.

Although the elevation of plasma leucine concentrations induced considerably lower concentrations of plasma isoleucine and valine, the mood states of the study participants determined by POMS were not affected by oral leucine at any dose, thus suggesting that the temporal profiles of plasma amino acids induced by leucine ingestion do not affect amino acid status in the brains of healthy men. This is consistent with the findings of a study of up to 1250 mg/kg BW/day of oral leucine to determine tolerable maximal intake (Elango et al. 2012). Mixtures of BCAAs are popular dietary supplements among athletes and are also used as medicines for patients with liver cirrhosis; many of the physiological effects of BCAAs can be attributed to the action of leucine. However, since information about the physiological effects of an altered plasma amino acid balance remains limited, leucine should not be ingested alone to avoid temporal disruption of the plasma BCAA balance. Mixtures of BCAAs can be recommended as a safe dietary sports supplement or medicine.

## Electronic supplementary material

Additional file 1: Table S1: Concentrations of plasma BCAAs, methionine, and AAAs in the leucine ingestion experiments. **Table S2.** Concentrations of plasma amino acids other than BCAAs, methionine, and AAAs in the leucine ingestion experiments. **Table S3.** Concentrations of plasma BCAAs, methionine, and AAAs in the isoleucine ingestion experimenst. **Table S4.** Concentrations of plasma amino acids other than BCAAs, methionine, and AAAs in the isoleucine ingestion experiments. **Table S5.** Concentrations of plasma BCAAs, methionine, and AAAs in the valine ingestion experiments. **Table S6.** Concentrations of plasma amino acids other than BCAAs, methionine, and AAAs in the valine ingestion experiments. **Table S7.** Concentrations of plasma BCAAs, methionine, and AAAs in the mixed BCAA ingestion experiments. **Table S8.** Concentrations of plasma amino acids other than BCAAs, methionine, and AAAs in the mixed BCAA ingestion experimensts. (DOC 1 MB)

Additional file 2: **Online Resource 1.** Concentrations of plasma components measured other than amino acids in the leucine ingestion experiments. **Online Resource 2.** Concentrations of plasma components measured other than amino acids in the isoleucine ingestion experiments. **Online Resource 3.** Concentrations of plasma components measured other than amino acids in the valine ingestion experiments. **Online Resource 4.** Concentrations of plasma components measured other than amino acids in the mixed BCAA ingestion experiments. (DOCX 357 KB)
