# Supplementary material for: Bolus ingestion of individual branched-chain amino acids alters plasma amino acid profiles in young healthy men
Source: Springerplus. 2014 Jan 17;3:35. doi: 10.1186/2193-1801-3-35 (PMC4320164; doi:10.1186/2193-1801-3-35)
Supplement: Supplementary file 1 — Additional file 1: Table S1: Concentrations of plasma BCAAs, methionine, and AAAs in the leucine ingestion experiments. Table S2. Concentrations of plasma amino acids other than BCAAs, methionine, and AAAs in the leucine ingestion experiments. Table S3. Concentrations of plasma BCAAs, methionine, and AAAs in the isoleucine ingestion experimenst. Table S4. Concentrations of plasma amino acids other than BCAAs, methionine, and AAAs in the isoleucine ingestion experiments. Table S5. Concentrations of plasma BCAAs, methionine, and AAAs in the valine ingestion experiments. Table S6. Concentrations of plasma amino acids other than BCAAs, methionine, and AAAs in the valine ingestion experiments. Table S7. Concentrations of plasma BCAAs, methionine, and AAAs in the mixed BCAA ingestion experiments. Table S8. Concentrations of plasma amino acids other than BCAAs, methionine, and AAAs in the mixed BCAA ingestion experimensts. (DOC 1 MB) [file 40064_2013_1453_MOESM1_ESM.doc]

**Table S1** Concentrations of plasma BCAAs, methionine, and AAAs in the leucine ingestion experiments

| Plasma amino acid | Leu ingested (mg/kg BW) | Time after ingestion (min) | | | | | | | | | | | P value |
| --- | --- | --- | --- | --- | --- | --- | --- | --- | --- | --- | --- | --- | --- |
|  |  | 0 | 15 | 30 | 45 | 60 | 75 | 90 | 105 | 120 | 180 | 240 |  |
| (μM) | | | | | | | | | | | | | |
| Leu | 0 | 128 ± 8 | 120 ± 8 | 112 ± 9 | 107 ± 7 | 105 ± 6 | 107 ± 5 | 106 ± 7 | 104 ± 6 | 105 ± 6 | 113 ± 3 | 120 ± 2 | NS |
|  | 10 | 132 ± 7 | 220 ± 32 | 220 ± 14 | 188 ± 13 | 164 ± 15 | 150 ± 9 | 145 ± 8 | 146 ± 8 | 142 ± 10 | 132 ± 9 | 134 ± 5 | <0.05 |
|  | 20 | 125 ± 7 | 316 ± 56 | 327 ± 33 | 261 ± 28 | 222 ± 18 | 195 ± 14 | 178 ± 11 | 164 ± 10 | 157 ± 10 | 138 ± 11 | 136 ± 8 | <0.05 |
|  | 30 | 137 ± 10 | 414 ± 48 | 436 ± 32 | 348 ± 36 | 301 ± 30 | 258 ± 23 | 232 ± 23 | 212 ± 18 | 201 ± 16 | 161 ± 20 | 147 ± 12 | <0.05 |
|  | 45 | 144 ± 8 | 604 ± 71 | 743 ± 51 | 606 ± 96 | 416 ± 53 | 345 ± 30 | 323 ± 28 | 290 ± 35 | 254 ± 38 | 192 ± 32 | 176 ± 16 | <0.05 |
|  | 60 | 143 ± 8 | 645 ± 92 | 807 ± 72 | 699 ± 87 | 576 ± 79 | 426 ± 62 | 353 ± 36 | 307 ± 28 | 274 ± 24 | 198 ± 29 | 176 ± 18 | <0.05 |
|  | 75 | 130 ± 3 | 432 ± 53 | 739 ± 79 | 708 ± 67 | 635 ± 57 | 551 ± 62 | 438 ± 57 | 374 ± 31 | 337 ± 22 | 231 ± 33 | 189 ± 23 | <0.05 |
|  | 90 | 135 ± 6 | 502 ± 71 | 785 ± 67 | 750 ± 71 | 672 ± 63 | 627 ± 71 | 534 ± 66 | 431 ± 68 | 355 ± 57 | 232 ± 50 | 188 ± 20 | <0.05 |
| Ile | 0 | 67 ± 6 | 62 ± 6 | 57 ± 6 | 54 ± 5 | 52 ± 5 | 54 ± 5 | 53 ± 5 | 51 ± 5 | 52 ± 5 | 54 ± 3 | 57 ± 3 | NS |
|  | 10 | 67 ± 4 | 61 ± 6 | 54 ± 5 | 46 ± 5 | 42 ± 5 | 40 ± 4 | 38 ± 3 | 39 ± 3 | 39 ± 4 | 39 ± 3 | 44 ± 3 | <0.05 |
|  | 20 | 66 ± 4 | 58 ± 5 | 51 ± 6 | 41 ± 6 | 35 ± 5 | 32 ± 3 | 30 ± 3 | 29 ± 3 | 28 ± 3 | 28 ± 3 | 32 ± 2 | <0.05 |
|  | 30 | 75 ± 6 | 67 ± 7 | 58 ± 7 | 47 ± 7 | 39 ± 6 | 33 ± 5 | 31 ± 5 | 29 ± 5 | 27 ± 4 | 26 ± 4 | 30 ± 2 | <0.05 |
|  | 45 | 79 ± 5 | 68 ± 6 | 58 ± 6 | 45 ± 6 | 35 ± 5 | 29 ± 4 | 26 ± 4 | 24 ± 3 | 23 ± 3 | 20 ± 2 | 26 ± 1 | <0.05 |
|  | 60 | 80 ± 4 | 71 ± 5 | 60 ± 5 | 46 ± 5 | 36 ± 4 | 27 ± 3 | 23 ± 3 | 21 ± 2 | 20 ± 2 | 16 ± 2 | 23 ± 1 | <0.05 |
|  | 75 | 66 ± 2 | 60 ± 3 | 53 ± 3 | 42 ± 3 | 34 ± 3 | 26 ± 3 | 22 ± 2 | 20 ± 3 | 18 ± 2 | 15 ± 2 | 20 ± 2 | <0.05 |
|  | 90 | 65 ± 4 | 56 ± 4 | 50 ± 4 | 40 ± 4 | 30 ± 4 | 22 ± 3 | 18 ± 2 | 16 ± 2 | 13 ± 2 | 16 ± 1 | 19 ± 1 | <0.05 |
| Val | 0 | 251 ± 11 | 243 ± 11 | 238 ± 11 | 233 ± 10 | 227 ± 9 | 230 ± 9 | 228 ± 11 | 224 ± 10 | 225 ± 10 | 227 ± 8 | 233 ± 8 | NS |
|  | 10 | 252 ± 10 | 242 ± 14 | 232 ± 15 | 219 ± 14 | 209 ± 14 | 201 ± 12 | 198 ± 11 | 201 ± 11 | 200 ± 11 | 196 ± 10 | 201 ± 6 | <0.05 |
|  | 20 | 239 ± 8 | 225 ± 11 | 216 ± 12 | 199 ± 13 | 186 ± 12 | 177 ± 11 | 174 ± 9 | 170 ± 10 | 168 ± 9 | 164 ± 9 | 166 ± 9 | <0.05 |
|  | 30 | 252 ± 18 | 241 ± 17 | 228 ± 17 | 209 ± 17 | 194 ± 16 | 181 ± 16 | 175 ± 15 | 171 ± 14 | 168 ± 14 | 156 ± 15 | 155 ± 11 | <0.05 |
|  | 45 | 280 ± 14 | 262 ± 16 | 247 ± 16 | 224 ± 17 | 204 ± 16 | 186 ± 15 | 180 ± 13 | 173 ± 13 | 168 ± 14 | 154 ± 13 | 157 ± 9 | <0.05 |
|  | 60 | 273 ± 13 | 264 ± 15 | 241 ± 14 | 217 ± 15 | 197 ± 14 | 179 ± 13 | 164 ± 12 | 157 ± 11 | 150 ± 11 | 135 ± 10 | 138 ± 6 | <0.05 |
|  | 75 | 253 ± 8 | 241 ± 9 | 231 ± 9 | 210 ± 10 | 193 ± 10 | 173 ± 9 | 161 ± 8 | 152 ± 11 | 146 ± 9 | 126 ± 8 | 125 ± 6 | <0.05 |
|  | 90 | 254 ± 11 | 237 ± 11 | 232 ± 11 | 212 ± 11 | 188 ± 11 | 167 ± 12 | 154 ± 11 | 141 ± 10 | 132 ± 8 | 118 ± 8 | 117 ± 5 | <0.05 |
| Met | 0 | 34 ± 2 | 32 ± 2 | 30 ± 2 | 29 ± 2 | 28 ± 2 | 28 ± 2 | 27 ± 2 | 26 ± 2 | 27 ± 2 | 27 ± 2 | 27 ± 1 | NS |
|  | 10 | 33 ± 1 | 31 ± 2 | 30 ± 1 | 27 ± 1 | 26 ± 1 | 26 ± 1 | 24 ± 1 | 26 ± 1 | 24 ± 2 | 23 ± 1 | 25 ± 2 | <0.05 |
|  | 20 | 30 ± 1 | 29 ± 1 | 27 ± 1 | 25 ± 1 | 23 ± 1 | 22 ± 1 | 21 ± 1 | 21 ± 1 | 20 ± 1 | 20 ± 1 | 21 ± 1 | <0.05 |
|  | 30 | 35 ± 2 | 32 ± 1 | 30 ± 1 | 29 ± 1 | 26 ± 1 | 24 ± 1 | 23 ± 1 | 23 ± 1 | 22 ± 1 | 20 ± 1 | 21 ± 1 | <0.05 |
|  | 45 | 34 ± 2 | 31 ± 1 | 28 ± 2 | 26 ± 2 | 24 ± 2 | 22 ± 2 | 21 ± 2 | 20 ± 1 | 19 ± 1 | 19 ± 1 | 20 ± 1 | <0.05 |
|  | 60 | 34 ± 2 | 31 ± 2 | 28 ± 2 | 26 ± 2 | 24 ± 2 | 22 ± 2 | 20 ± 1 | 20 ± 2 | 19 ± 2 | 17 ± 1 | 18 ± 1 | <0.05 |
|  | 75 | 31 ± 2 | 31 ± 2 | 29 ± 2 | 27 ± 2 | 26 ± 2 | 23 ± 2 | 22 ± 2 | 22 ± 2 | 20 ± 1 | 18 ± 1 | 18 ± 1 | <0.05 |
|  | 90 | 31 ± 2 | 29 ± 1 | 28 ± 2 | 27 ± 2 | 25 ± 2 | 22 ± 2 | 21 ± 2 | 19 ± 2 | 18 ± 1 | 17 ± 1 | 18 ± 2 | <0.05 |
| Phe | 0 | 68 ± 2 | 67 ± 3 | 63 ± 2 | 60 ± 3 | 59 ± 3 | 60 ± 2 | 58 ± 2 | 57 ± 2 | 58 ± 3 | 58 ± 2 | 61 ± 2 | NS |
|  | 10 | 63 ± 2 | 60 ± 3 | 56 ± 2 | 52 ± 2 | 53 ± 1 | 50 ± 2 | 49 ± 2 | 48 ± 1 | 49 ± 2 | 48 ± 2 | 51 ± 2 | <0.05 |
|  | 20 | 61 ± 2 | 60 ± 3 | 56 ± 3 | 49 ± 4 | 48 ± 4 | 45 ± 3 | 44 ± 2 | 44 ± 3 | 46 ± 2 | 43 ± 3 | 44 ± 2 | <0.05 |
|  | 30 | 60 ± 3 | 58 ± 2 | 54 ± 3 | 49 ± 3 | 45 ± 3 | 44 ± 3 | 41 ± 3 | 40 ± 2 | 39 ± 2 | 37 ± 2 | 41 ± 2 | <0.05 |
|  | 45 | 67 ± 3 | 63 ± 2 | 56 ± 3 | 50 ± 2 | 47 ± 3 | 43 ± 2 | 43 ± 2 | 41 ± 1 | 40 ± 2 | 39 ± 1 | 46 ± 1 | <0.05 |
|  | 60 | 65 ± 2 | 62 ± 2 | 55 ± 2 | 49 ± 2 | 44 ± 2 | 42 ± 2 | 38 ± 2 | 38 ± 2 | 37 ± 1 | 37 ± 1 | 42 ± 2 | <0.05 |
|  | 75 | 61 ± 1 | 59 ± 1 | 56 ± 1 | 52 ± 1 | 48 ± 2 | 44 ± 2 | 42 ± 2 | 41 ± 2 | 40 ± 1 | 38 ± 1 | 41 ± 1 | <0.05 |
|  | 90 | 63 ± 2 | 59 ± 2 | 57 ± 2 | 53 ± 2 | 48 ± 2 | 45 ± 2 | 41 ± 2 | 40 ± 2 | 39 ± 1 | 39 ± 1 | 43 ± 2 | <0.05 |
| Trp | 0 | 59 ± 5 | 56 ± 5 | 53 ± 5 | 53 ± 5 | 51 ± 5 | 51 ± 4 | 50 ± 4 | 49 ± 4 | 48 ± 3 | 46 ± 3 | 48 ± 4 | NS |
|  | 10 | 59 ± 3 | 55 ± 4 | 53 ± 3 | 51 ± 3 | 50 ± 3 | 47 ± 3 | 47 ± 3 | 47 ± 3 | 47 ± 4 | 46 ± 4 | 46 ± 3 | NS |
|  | 20 | 64 ± 2 | 62 ± 3 | 60 ± 2 | 61 ± 3 | 57 ± 3 | 58 ± 4 | 54 ± 3 | 55 ± 4 | 52 ± 4 | 49 ± 4 | 50 ± 4 | NS |
|  | 30 | 57 ± 4 | 54 ± 4 | 52 ± 4 | 49 ± 4 | 46 ± 3 | 44 ± 3 | 42 ± 4 | 43 ± 4 | 42 ± 4 | 40 ± 4 | 42 ± 2 | NS |
|  | 45 | 54 ± 4 | 50 ± 4 | 45 ± 5 | 44 ± 3 | 44 ± 4 | 42 ± 4 | 40 ± 3 | 40 ± 3 | 39 ± 3 | 38 ± 3 | 39 ± 3 | NS |
|  | 60 | 54 ± 4 | 51 ± 4 | 46 ± 4 | 45 ± 4 | 40 ± 3 | 39 ± 4 | 37 ± 3 | 38 ± 3 | 39 ± 4 | 36 ± 3 | 40 ± 3 | <0.05 |
|  | 75 | 59 ± 2 | 57 ± 3 | 54 ± 3 | 53 ± 4 | 53 ± 2 | 47 ± 3 | 47 ± 3 | 45 ± 3 | 44 ± 3 | 43 ± 3 | 45 ± 2 | <0.05 |
|  | 90 | 62 ± 2 | 56 ± 3 | 54 ± 3 | 54 ± 2 | 51 ± 2 | 47 ± 3 | 46 ± 3 | 44 ± 3 | 47 ± 2 | 44 ± 2 | 47 ± 1 | <0.05 |
| Tyr | 0 | 78 ± 6 | 75 ± 7 | 71 ± 6 | 68 ± 6 | 67 ± 6 | 67 ± 5 | 65 ± 6 | 63 ± 6 | 64 ± 5 | 63 ± 4 | 64 ± 3 | NS |
|  | 10 | 75 ± 4 | 71 ± 6 | 66 ± 5 | 63 ± 4 | 60 ± 5 | 58 ± 4 | 56 ± 4 | 57 ± 4 | 55 ± 4 | 55 ± 4 | 56 ± 3 | <0.05 |
|  | 20 | 75 ± 4 | 70 ± 5 | 68 ± 5 | 63 ± 5 | 60 ± 4 | 56 ± 4 | 56 ± 3 | 55 ± 3 | 55 ± 3 | 54 ± 3 | 55 ± 3 | <0.05 |
|  | 30 | 77 ± 4 | 73 ± 4 | 70 ± 4 | 65 ± 3 | 61 ± 3 | 59 ± 3 | 55 ± 3 | 54 ± 3 | 54 ± 3 | 49 ± 4 | 48 ± 2 | <0.05 |
|  | 45 | 80 ± 5 | 73 ± 5 | 69 ± 5 | 64 ± 5 | 60 ± 5 | 56 ± 5 | 55 ± 4 | 52 ± 4 | 52 ± 5 | 48 ± 4 | 50 ± 3 | <0.05 |
|  | 60 | 82 ± 5 | 76 ± 5 | 71 ± 5 | 67 ± 5 | 62 ± 5 | 58 ± 5 | 53 ± 4 | 53 ± 4 | 51 ± 4 | 48 ± 3 | 50 ± 2 | <0.05 |
|  | 75 | 74 ± 3 | 72 ± 3 | 69 ± 3 | 66 ± 3 | 62 ± 3 | 57 ± 2 | 55 ± 2 | 53 ± 2 | 52 ± 2 | 46 ± 2 | 45 ± 1 | <0.05 |
|  | 90 | 77 ± 4 | 72 ± 3 | 71 ± 4 | 67 ± 5 | 63 ± 4 | 59 ± 5 | 56 ± 4 | 52 ± 4 | 51 ± 4 | 48 ± 3 | 48 ± 3 | <0.05 |

Values are means ± SE. *P* values were calculated for changes over time within each dose of leucine ingestion by one-way rmANOVA. NS stands for not significant.

**Table S2** Concentrations of plasma amino acids other than BCAAs, methionine, and AAAs in the leucine ingestion experiments

| Plasma amino acid | Leu ingested (mg/kg BW) | Time after ingestion (min) | | | | | | | | | | | P value |
| --- | --- | --- | --- | --- | --- | --- | --- | --- | --- | --- | --- | --- | --- |
|  |  | 0 | 15 | 30 | 45 | 60 | 75 | 90 | 105 | 120 | 180 | 240 |  |
| (μM) | | | | | | | | | | | | | |
| Ala | 0 | 375 ± 25 | 390 ± 25 | 389 ± 26 | 377 ± 23 | 359 ± 26 | 363 ± 21 | 351 ± 22 | 339 ± 22 | 333 ± 22 | 326 ± 15 | 316 ± 13 | NS |
|  | 10 | 386 ± 26 | 379 ± 25 | 367 ± 21 | 358 ± 22 | 348 ± 20 | 331 ± 23 | 326 ± 19 | 334 ± 19 | 321 ± 26 | 318 ± 23 | 310 ± 16 | NS |
|  | 20 | 403 ± 16 | 378 ± 21 | 365 ± 23 | 348 ± 25 | 326 ± 24 | 312 ± 18 | 317 ± 14 | 317 ± 21 | 318 ± 20 | 306 ± 21 | 308 ± 18 | <0.05 |
|  | 30 | 416 ± 24 | 395 ± 22 | 387 ± 12 | 362 ± 15 | 344 ± 7 | 345 ± 8 | 332 ± 8 | 340 ± 9 | 338 ± 10 | 322 ± 18 | 306 ± 22 | <0.05 |
|  | 45 | 433 ± 30 | 389 ± 30 | 364 ± 21 | 341 ± 17 | 346 ± 19 | 333 ± 23 | 336 ± 22 | 323 ± 15 | 330 ± 21 | 342 ± 20 | 351 ± 27 | NS |
|  | 60 | 423 ± 21 | 389 ± 24 | 352 ± 17 | 329 ± 16 | 319 ± 17 | 322 ± 20 | 304 ± 20 | 311 ± 18 | 306 ± 18 | 328 ± 23 | 327 ± 24 | <0.05 |
|  | 75 | 399 ± 22 | 383 ± 19 | 359 ± 21 | 352 ± 19 | 354 ± 22 | 337 ± 17 | 339 ± 20 | 348 ± 25 | 342 ± 24 | 318 ± 23 | 300 ± 23 | NS |
|  | 90 | 386 ± 21 | 359 ± 21 | 353 ± 17 | 345 ± 18 | 337 ± 19 | 324 ± 21 | 321 ± 23 | 307 ± 26 | 311 ± 21 | 292 ± 23 | 292 ± 20 | NS |
| Arg | 0 | 115 ± 8 | 115 ± 9 | 107 ± 7 | 103 ± 8 | 101 ± 8 | 102 ± 8 | 102 ± 7 | 97 ± 8 | 98 ± 8 | 98 ± 8 | 101 ± 5 | NS |
|  | 10 | 109 ± 7 | 107 ± 9 | 102 ± 6 | 102 ± 7 | 99 ± 7 | 96 ± 7 | 96 ± 9 | 97 ± 8 | 95 ± 7 | 92 ± 8 | 92 ± 6 | NS |
|  | 20 | 106 ± 8 | 99 ± 7 | 103 ± 7 | 101 ± 8 | 94 ± 6 | 91 ± 6 | 92 ± 7 | 91 ± 8 | 91 ± 9 | 89 ± 8 | 88 ± 7 | NS |
|  | 30 | 114 ± 6 | 109 ± 7 | 111 ± 7 | 113 ± 7 | 106 ± 5 | 107 ± 6 | 102 ± 5 | 103 ± 6 | 103 ± 6 | 96 ± 4 | 88 ± 3 | NS |
|  | 45 | 115 ± 6 | 110 ± 3 | 115 ± 6 | 114 ± 4 | 114 ± 8 | 109 ± 7 | 106 ± 7 | 104 ± 5 | 99 ± 4 | 93 ± 4 | 94 ± 6 | NS |
|  | 60 | 115 ± 10 | 120 ± 9 | 119 ± 9 | 115 ± 9 | 115 ± 10 | 113 ± 10 | 107 ± 9 | 107 ± 10 | 101 ± 9 | 96 ± 8 | 93 ± 8 | NS |
|  | 75 | 114 ± 5 | 124 ± 8 | 131 ± 11 | 134 ± 8 | 130 ± 7 | 123 ± 6 | 121 ± 6 | 122 ± 5 | 117 ± 4 | 97 ± 5 | 92 ± 3 | <0.05 |
|  | 90 | 122 ± 6 | 120 ± 11 | 138 ± 15 | 138 ± 14 | 135 ± 13 | 130 ± 13 | 124 ± 13 | 122 ± 11 | 111 ± 12 | 101 ± 10 | 96 ± 8 | NS |
| Asn | 0 | 49 ± 3 | 48 ± 3 | 48 ± 3 | 45 ± 3 | 46 ± 4 | 45 ± 3 | 46 ± 4 | 44 ± 3 | 43 ± 3 | 43 ± 3 | 45 ± 3 | NS |
|  | 10 | 44 ± 3 | 43 ± 3 | 41 ± 2 | 40 ± 3 | 40 ± 3 | 39 ± 3 | 38 ± 3 | 39 ± 3 | 41 ± 3 | 38 ± 2 | 38 ± 2 | NS |
|  | 20 | 46 ± 3 | 43 ± 2 | 43 ± 2 | 40 ± 2 | 38 ± 2 | 38 ± 3 | 39 ± 3 | 37 ± 2 | 39 ± 3 | 37 ± 2 | 39 ± 3 | NS |
|  | 30 | 47 ± 2 | 44 ± 2 | 42 ± 1 | 39 ± 1 | 37 ± 2 | 37 ± 3 | 36 ± 3 | 38 ± 3 | 37 ± 2 | 36 ± 2 | 36 ± 2 | <0.05 |
|  | 45 | 49 ± 3 | 45 ± 1 | 43 ± 2 | 39 ± 3 | 38 ± 3 | 36 ± 3 | 38 ± 4 | 37 ± 2 | 39 ± 2 | 37 ± 2 | 41 ± 3 | NS |
|  | 60 | 48 ± 3 | 46 ± 3 | 42 ± 2 | 39 ± 2 | 37 ± 3 | 37 ± 3 | 35 ± 2 | 37 ± 3 | 35 ± 2 | 36 ± 3 | 38 ± 3 | <0.05 |
|  | 75 | 51 ± 3 | 50 ± 3 | 48 ± 3 | 47 ± 3 | 45 ± 2 | 42 ± 2 | 43 ± 3 | 44 ± 2 | 42 ± 2 | 41 ± 2 | 41 ± 2 | NS |
|  | 90 | 50 ± 3 | 47 ± 2 | 46 ± 3 | 44 ± 3 | 41 ± 3 | 41 ± 3 | 40 ± 3 | 40 ± 3 | 40 ± 3 | 37 ± 3 | 37 ± 4 | NS |
| Asp | 0 | 0.6 ± 0.5 | 0.8 ± 0.5 | 0.0 ± 0.7 | 0.6 ± 0.0 | 0.6 ± 0.5 | 0.7 ± 0.5 | 0.7 ± 0.6 | 0.6 ± 0.6 | 1.4 ± 0.7 | 0.6 ± 0.7 | 0.7 ± 0.6 | NS |
|  | 10 | 0.0 ± 0.0 | 0.0 ± 0.0 | 0.0 ± 0.0 | 0.0 ± 0.0 | 0.0 ± 0.0 | 0.0 ± 0.0 | 0.0 ± 0.0 | 0.6 ± 0.6 | 0.6 ± 0.6 | 0.8 ± 0.7 | 0.7 ± 0.6 | NS |
|  | 20 | 0.7 ± 0.7 | 0.0 ± 0.0 | 0.0 ± 0.0 | 0.0 ± 0.0 | 0.7 ± 0.6 | 0.0 ± 0.0 | 0.9 ± 0.0 | 0.6 ± 0.8 | 0.0 ± 0.0 | 0.7 ± 0.6 | 0.0 ± 0.0 | NS |
|  | 30 | 1.0 ± 0.9 | 2.0 ± 0.8 | 0.8 ± 0.8 | 1.4 ± 0.6 | 0.7 ± 0.7 | 0.7 ± 0.7 | 0.8 ± 0.7 | 1.3 ± 0.7 | 0.7 ± 0.6 | 0.6 ± 0.6 | 0.6 ± 0.6 | NS |
|  | 45 | 0.6 ± 0.6 | 0.8 ± 0.6 | 0.0 ± 0.7 | 0.6 ± 0.6 | 0.0 ± 0.0 | 0.0 ± 0.0 | 0.0 ± 0.0 | 0.0 ± 0.0 | 0.6 ± 0.0 | 1.5 ± 0.8 | 0.6 ± 0.0 | NS |
|  | 60 | 0.0 ± 0.0 | 1.3 ± 0.7 | 0.6 ± 0.0 | 0.0 ± 0.5 | 1.2 ± 0.6 | 0.7 ± 0.7 | 0.0 ± 0.0 | 0.0 ± 0.0 | 0.0 ± 0.0 | 1.5 ± 0.6 | 0.0 ± 0.8 | NS |
|  | 75 | 1.8 ± 0.4 | 1.0 ± 0.5 | 1.7 ± 0.4 | 1.4 ± 0.5 | 1.5 ± 0.3 | 0.6 ± 0.4 | 1.1 ± 0.4 | 1.3 ± 0.5 | 1.1 ± 0.4 | 1.0 ± 0.4 | 0.7 ± 0.4 | NS |
|  | 90 | 1.5 ± 0.4 | 1.2 ± 0.3 | 0.6 ± 0.3 | 0.8 ± 0.4 | 0.3 ± 0.3 | 0.0 ± 0.0 | 0.8 ± 0.4 | 1.4 ± 0.4 | 0.7 ± 0.4 | 1.1 ± 0.4 | 0.3 ± 0.4 | NS |
| Cys | 0 | 54 ± 3 | 53 ± 3 | 54 ± 3 | 54 ± 3 | 53 ± 3 | 53 ± 3 | 54 ± 3 | 54 ± 3 | 53 ± 3 | 52 ± 3 | 51 ± 3 | NS |
|  | 10 | 59 ± 4 | 58 ± 4 | 58 ± 3 | 56 ± 3 | 55 ± 3 | 54 ± 3 | 53 ± 3 | 54 ± 2 | 55 ± 4 | 53 ± 3 | 55 ± 3 | NS |
|  | 20 | 58 ± 3 | 57 ± 3 | 56 ± 3 | 54 ± 3 | 55 ± 3 | 55 ± 3 | 54 ± 3 | 53 ± 3 | 52 ± 3 | 52 ± 3 | 51 ± 3 | NS |
|  | 30 | 58 ± 3 | 57 ± 3 | 55 ± 3 | 54 ± 3 | 54 ± 3 | 53 ± 3 | 53 ± 3 | 52 ± 3 | 52 ± 3 | 51 ± 3 | 51 ± 2 | NS |
|  | 45 | 65 ± 4 | 63 ± 4 | 61 ± 4 | 61 ± 3 | 58 ± 3 | 57 ± 3 | 57 ± 3 | 59 ± 3 | 59 ± 3 | 56 ± 4 | 55 ± 3 | NS |
|  | 60 | 61 ± 3 | 60 ± 3 | 56 ± 3 | 56 ± 3 | 56 ± 3 | 54 ± 3 | 54 ± 2 | 54 ± 3 | 54 ± 3 | 52 ± 3 | 53 ± 3 | NS |
|  | 75 | 56 ± 2 | 53 ± 3 | 52 ± 2 | 51 ± 2 | 50 ± 3 | 50 ± 3 | 49 ± 2 | 48 ± 2 | 49 ± 2 | 49 ± 2 | 48 ± 2 | NS |
|  | 90 | 57 ± 2 | 53 ± 3 | 53 ± 2 | 51 ± 3 | 51 ± 2 | 50 ± 2 | 50 ± 2 | 50 ± 2 | 48 ± 2 | 50 ± 1 | 50 ± 2 | NS |
| Gln | 0 | 612 ± 21 | 590 ± 29 | 587 ± 28 | 577 ± 30 | 568 ± 34 | 578 ± 29 | 562 ± 29 | 543 ± 34 | 561 ± 35 | 597 ± 32 | 611 ± 20 | NS |
|  | 10 | 625 ± 22 | 596 ± 27 | 580 ± 21 | 596 ± 21 | 577 ± 21 | 575 ± 20 | 574 ± 25 | 592 ± 21 | 590 ± 32 | 612 ± 25 | 626 ± 23 | NS |
|  | 20 | 582 ± 18 | 559 ± 17 | 587 ± 20 | 580 ± 27 | 553 ± 25 | 528 ± 14 | 547 ± 16 | 565 ± 13 | 583 ± 24 | 617 ± 18 | 620 ± 16 | NS |
|  | 30 | 604 ± 23 | 578 ± 31 | 594 ± 25 | 603 ± 34 | 584 ± 25 | 579 ± 27 | 563 ± 33 | 595 ± 39 | 599 ± 35 | 602 ± 34 | 601 ± 24 | NS |
|  | 45 | 610 ± 95 | 81 ± 11 | 611 ± 20 | 594 ± 13 | 613 ± 15 | 584 ± 20 | 587 ± 18 | 574 ± 75 | 83 ± 13 | 611 ± 18 | 661 ± 17 | <0.05 |
|  | 60 | 593 ± 23 | 582 ± 30 | 592 ± 23 | 583 ± 21 | 576 ± 22 | 589 ± 24 | 558 ± 32 | 580 ± 18 | 567 ± 18 | 604 ± 29 | 626 ± 20 | NS |
|  | 75 | 624 ± 11 | 659 ± 22 | 680 ± 21 | 703 ± 23 | 709 ± 25 | 679 ± 26 | 684 ± 20 | 700 ± 18 | 687 ± 10 | 684 ± 96 | 76 ± 10 | NS |
|  | 90 | 611 ± 15 | 628 ± 27 | 679 ± 35 | 688 ± 34 | 677 ± 34 | 671 ± 33 | 681 ± 37 | 644 ± 42 | 668 ± 33 | 642 ± 31 | 659 ± 18 | NS |
| Glu | 0 | 34 ± 9 | 39 ± 10 | 42 ± 11 | 42 ± 9 | 48 ± 9 | 46 ± 10 | 49 ± 9 | 51 ± 9 | 50 ± 9 | 43 ± 10 | 40 ± 10 | NS |
|  | 10 | 40 ± 6 | 43 ± 6 | 47 ± 5 | 45 ± 5 | 44 ± 7 | 45 ± 7 | 47 ± 7 | 45 ± 7 | 53 ± 6 | 47 ± 9 | 45 ± 9 | NS |
|  | 20 | 47 ± 7 | 47 ± 8 | 45 ± 7 | 46 ± 6 | 53 ± 7 | 55 ± 5 | 51 ± 7 | 47 ± 7 | 44 ± 9 | 41 ± 9 | 39 ± 6 | NS |
|  | 30 | 45 ± 7 | 48 ± 5 | 42 ± 6 | 42 ± 6 | 51 ± 7 | 42 ± 7 | 48 ± 7 | 44 ± 9 | 45 ± 7 | 42 ± 7 | 41 ± 5 | NS |
|  | 45 | 56 ± 8 | 64 ± 7 | 63 ± 10 | 65 ± 8 | 53 ± 10 | 55 ± 9 | 56 ± 8 | 61 ± 6 | 61 ± 7 | 53 ± 8 | 47 ± 6 | NS |
|  | 60 | 49 ± 8 | 57 ± 8 | 57 ± 7 | 58 ± 7 | 56 ± 6 | 50 ± 8 | 56 ± 6 | 53 ± 6 | 54 ± 8 | 46 ± 8 | 46 ± 8 | NS |
|  | 75 | 36 ± 4 | 26 ± 5 | 30 ± 5 | 28 ± 4 | 28 ± 4 | 32 ± 4 | 33 ± 5 | 29 ± 4 | 31 ± 3 | 32 ± 3 | 29 ± 3 | NS |
|  | 90 | 36 ± 5 | 28 ± 7 | 32 ± 5 | 30 ± 6 | 29 ± 5 | 29 ± 4 | 33 ± 6 | 37 ± 7 | 34 ± 5 | 38 ± 5 | 28 ± 7 | NS |
| (Table S2-2) | | | | | | | | | | | | | |
| (μM) | | | | | | | | | | | | | |
| Gly | 0 | 268 ± 6 | 263 ± 8 | 261 ± 7 | 258 ± 9 | 254 ± 9 | 258 ± 8 | 255 ± 7 | 247 ± 9 | 251 ± 10 | 251 ± 8 | 253 ± 6 | NS |
|  | 10 | 251 ± 13 | 238 ± 13 | 234 ± 12 | 235 ± 12 | 230 ± 13 | 228 ± 14 | 226 ± 14 | 234 ± 15 | 231 ± 11 | 232 ± 14 | 232 ± 10 | NS |
|  | 20 | 240 ± 13 | 227 ± 13 | 228 ± 13 | 223 ± 14 | 221 ± 12 | 215 ± 10 | 220 ± 82 | 22 ± 10 | 224 ± 11 | 229 ± 12 | 230 ± 8 | NS |
|  | 30 | 275 ± 5 | 254 ± 7 | 250 ± 5 | 247 ± 7 | 244 ± 5 | 244 ± 7 | 240 ± 6 | 246 ± 8 | 250 ± 6 | 244 ± 6 | 238 ± 6 | NS |
|  | 45 | 262 ± 4 | 243 ± 5 | 238 ± 4 | 230 ± 4 | 233 ± 7 | 230 ± 7 | 230 ± 6 | 228 ± 3 | 230 ± 4 | 233 ± 3 | 245 ± 7 | <0.05 |
|  | 60 | 257 ± 9 | 244 ± 10 | 225 ± 6 | 218 ± 8 | 218 ± 9 | 220 ± 9 | 215 ± 8 | 219 ± 7 | 218 ± 7 | 226 ± 9 | 229 ± 11 | NS |
|  | 75 | 275 ± 10 | 270 ± 10 | 257 ± 9 | 252 ± 10 | 251 ± 13 | 243 ± 13 | 247 ± 10 | 251 ± 14 | 250 ± 12 | 239 ± 9 | 236 ± 9 | NS |
|  | 90 | 252 ± 4 | 241 ± 6 | 237 ± 8 | 231 ± 8 | 226 ± 7 | 222 ± 7 | 223 ± 7 | 217 ± 7 | 220 ± 6 | 218 ± 7 | 218 ± 6 | <0.05 |
| His | 0 | 93 ± 3 | 88 ± 4 | 88 ± 2 | 86 ± 3 | 85 ± 3 | 86 ± 3 | 86 ± 3 | 84 ± 3 | 86 ± 3 | 88 ± 3 | 89 ± 1 | NS |
|  | 10 | 93 ± 2 | 89 ± 4 | 88 ± 4 | 89 ± 2 | 84 ± 3 | 84 ± 3 | 83 ± 3 | 85 ± 3 | 86 ± 3 | 87 ± 3 | 88 ± 2 | NS |
|  | 20 | 81 ± 5 | 79 ± 5 | 80 ± 5 | 76 ± 6 | 72 ± 5 | 72 ± 4 | 73 ± 4 | 74 ± 4 | 76 ± 4 | 77 ± 4 | 78 ± 4 | NS |
|  | 30 | 91 ± 2 | 87 ± 3 | 86 ± 3 | 85 ± 2 | 82 ± 1 | 80 ± 1 | 78 ± 1 | 82 ± 2 | 81 ± 2 | 82 ± 1 | 82 ± 1 | <0.05 |
|  | 45 | 97 ± 3 | 91 ± 4 | 92 ± 2 | 89 ± 2 | 88 ± 3 | 82 ± 4 | 85 ± 3 | 83 ± 2 | 84 ± 3 | 88 ± 2 | 91 ± 1 | <0.05 |
|  | 60 | 91 ± 3 | 89 ± 3 | 87 ± 2 | 83 ± 2 | 81 ± 3 | 80 ± 3 | 78 ± 2 | 79 ± 2 | 79 ± 2 | 83 ± 1 | 86 ± 2 | <0.05 |
|  | 75 | 90 ± 3 | 93 ± 2 | 91 ± 2 | 89 ± 1 | 89 ± 1 | 86 ± 2 | 84 ± 1 | 85 ± 1 | 84 ± 1 | 85 ± 1 | 82 ± 2 | <0.05 |
|  | 90 | 93 ± 2 | 91 ± 2 | 96 ± 2 | 95 ± 2 | 89 ± 3 | 86 ± 3 | 86 ± 2 | 83 ± 3 | 84 ± 2 | 82 ± 3 | 85 ± 2 | <0.05 |
| Lys | 0 | 214 ± 8 | 209 ± 11 | 205 ± 9 | 201 ± 10 | 196 ± 11 | 200 ± 9 | 197 ± 9 | 193 ± 10 | 194 ± 10 | 195 ± 9 | 198 ± 6 | NS |
|  | 10 | 211 ± 10 | 206 ± 11 | 199 ± 9 | 199 ± 10 | 194 ± 10 | 190 ± 9 | 190 ± 11 | 195 ± 10 | 193 ± 11 | 190 ± 12 | 189 ± 10 | NS |
|  | 20 | 193 ± 9 | 186 ± 8 | 189 ± 8 | 185 ± 10 | 178 ± 7 | 174 ± 7 | 178 ± 9 | 179 ± 10 | 180 ± 11 | 178 ± 10 | 178 ± 12 | NS |
|  | 30 | 208 ± 11 | 201 ± 13 | 204 ± 14 | 202 ± 14 | 196 ± 13 | 196 ± 14 | 192 ± 12 | 196 ± 13 | 195 ± 12 | 185 ± 10 | 180 ± 8 | NS |
|  | 45 | 218 ± 8 | 207 ± 7 | 211 ± 12 | 206 ± 10 | 212 ± 13 | 204 ± 12 | 206 ± 13 | 198 ± 9 | 198 ± 9 | 192 ± 8 | 191 ± 8 | NS |
|  | 60 | 220 ± 15 | 217 ± 14 | 215 ± 15 | 210 ± 16 | 209 ± 18 | 211 ± 18 | 201 ± 16 | 203 ± 16 | 199 ± 15 | 191 ± 15 | 188 ± 15 | NS |
|  | 75 | 210 ± 12 | 220 ± 15 | 226 ± 15 | 233 ± 14 | 232 ± 14 | 219 ± 13 | 218 ± 13 | 215 ± 11 | 210 ± 10 | 189 ± 6 | 178 ± 5 | NS |
|  | 90 | 226 ± 6 | 229 ± 10 | 246 ± 15 | 250 ± 17 | 243 ± 15 | 235 ± 14 | 230 ± 15 | 216 ± 14 | 214 ± 13 | 190 ± 11 | 183 ± 11 | <0.05 |
| Pro | 0 | 147 ± 8 | 150 ± 8 | 144 ± 6 | 144 ± 7 | 138 ± 7 | 143 ± 7 | 141 ± 9 | 138 ± 10 | 134 ± 8 | 130 ± 7 | 126 ± 6 | NS |
|  | 10 | 161 ± 8 | 157 ± 9 | 150 ± 7 | 147 ± 6 | 141 ± 7 | 137 ± 7 | 135 ± 8 | 139 ± 8 | 138 ± 6 | 134 ± 8 | 133 ± 7 | NS |
|  | 20 | 133 ± 5 | 132 ± 6 | 131 ± 8 | 128 ± 12 | 125 ± 11 | 121 ± 10 | 121 ± 8 | 119 ± 9 | 120 ± 9 | 118 ± 10 | 120 ± 9 | NS |
|  | 30 | 157 ± 10 | 147 ± 9 | 144 ± 7 | 134 ± 8 | 133 ± 8 | 132 ± 8 | 131 ± 7 | 128 ± 7 | 135 ± 7 | 128 ± 9 | 123 ± 10 | NS |
|  | 45 | 175 ± 15 | 160 ± 14 | 150 ± 12 | 144 ± 12 | 141 ± 15 | 135 ± 14 | 138 ± 13 | 139 ± 10 | 142 ± 12 | 138 ± 12 | 142 ± 12 | NS |
|  | 60 | 172 ± 11 | 159 ± 10 | 142 ± 10 | 136 ± 10 | 132 ± 12 | 132 ± 11 | 125 ± 12 | 128 ± 12 | 126 ± 11 | 129 ± 13 | 130 ± 13 | NS |
|  | 75 | 148 ± 10 | 141 ± 9 | 133 ± 8 | 129 ± 9 | 129 ± 10 | 121v7 | 121 ± 10 | 125 ± 10 | 126 ± 10 | 117 ± 11 | 116 ± 10 | NS |
|  | 90 | 152 ± 13 | 140 ± 11 | 138v10 | 130 ± 12 | 128 ± 12 | 123 ± 12 | 121 ± 10 | 119 ± 10 | 120 ± 11 | 115 ± 12 | 114 ± 12 | NS |
| Ser | 0 | 125 ± 8 | 126 ± 8 | 125 ± 8 | 121 ± 9 | 120 ± 8 | 121 ± 8 | 123 ± 8 | 120 ± 8 | 122 ± 8 | 122 ± 7 | 122 ± 7 | NS |
|  | 10 | 125 ± 10 | 120 ± 8 | 117 ± 8 | 116 ± 9 | 112 ± 9 | 111 ± 9 | 112 ± 8 | 114 ± 10 | 117 ± 9 | 114 ± 8 | 116 ± 8 | NS |
|  | 20 | 119 ± 10 | 113 ± 8 | 111 ± 8 | 106 ± 8 | 107 ± 7 | 106 ± 8 | 106 ± 8 | 104 ± 7 | 103 ± 6 | 105 ± 7 | 103 ± 6 | NS |
|  | 30 | 128 ± 11 | 123 ± 8 | 116 ± 7 | 112 ± 8 | 112 ± 8 | 108 ± 9 | 109 ± 9 | 109 ± 9 | 109 ± 10 | 109 ± 10 | 109 ± 9 | NS |
|  | 45 | 131 ± 11 | 126 ± 9 | 120 ± 9 | 117 ± 10 | 112 ± 10 | 111 ± 11 | 112 ± 10 | 113 ± 9 | 114 ± 9 | 112 ± 9 | 112 ± 9 | NS |
|  | 60 | 121 ± 12 | 121 ± 14 | 109 ± 10 | 105 ± 10 | 104 ± 10 | 102 ± 9 | 100 ± 9 | 101 ± 9 | 101 ± 9 | 101 ± 10 | 103 ± 9 | NS |
|  | 75 | 123 ± 10 | 120 ± 11 | 117 ± 9 | 112 ± 10 | 109 ± 10 | 107 ± 10 | 106 ± 8 | 105 ± 10 | 103 ± 9 | 103 ± 8 | 103 ± 8 | NS |
|  | 90 | 120 ± 8 | 113 ± 5 | 113 ± 7 | 107 ± 6 | 103 ± 6 | 100 ± 6 | 100 ± 6 | 100 ± 6 | 98 ± 7 | 102 ± 8 | 98 ± 6 | NS |
| Thr | 0 | 151 ± 10 | 148 ± 10 | 146 ± 10 | 141 ± 10 | 137 ± 11 | 139 ± 11 | 138 ± 11 | 135 ± 10 | 137 ± 11 | 138 ± 10 | 140 ± 9 | NS |
|  | 10 | 144 ± 9 | 136 ± 11 | 132 ± 10 | 128 ± 10 | 126 ± 11 | 122 ± 11 | 122 ± 11 | 125 ± 11 | 125 ± 10 | 124 ± 11 | 125 ± 10 | NS |
|  | 20 | 129 ± 9 | 120 ± 9 | 117 ± 9 | 112 ± 10 | 108 ± 9 | 105 ± 9 | 107 ± 9 | 108 ± 9 | 108 ± 8 | 112 ± 10 | 112 ± 8 | NS |
|  | 30 | 153 ± 10 | 144 ± 8 | 138 ± 8 | 132 ± 8 | 128 ± 8 | 127 ± 9 | 124 ± 9 | 126 ± 8 | 127 ± 8 | 125 ± 8 | 124 ± 8 | NS |
|  | 45 | 147 ± 7 | 135 ± 5 | 130 ± 6 | 123 ± 6 | 121 ± 8 | 117 ± 8 | 119 ± 8 | 116 ± 7 | 117 ± 7 | 119 ± 7 | 124 ± 8 | NS |
|  | 60 | 143 ± 14 | 135 ± 12 | 124 ± 12 | 117 ± 12 | 114 ± 13 | 112 ± 13 | 107 ± 12 | 109 ± 12 | 109 ± 12 | 112 ± 13 | 114 ± 13 | NS |
|  | 75 | 145 ± 9 | 142 ± 8 | 136 ± 8 | 131 ± 6 | 128 ± 8 | 122 ± 6 | 122 ± 7 | 123 ± 7 | 121 ± 6 | 117 ± 5 | 116 ± 5 | NS |
|  | 90 | 138 ± 12 | 130 ± 10 | 129 ± 12 | 124 ± 12 | 118 ± 11 | 114 ± 11 | 113 ± 12 | 109 ± 12 | 109 ± 12 | 108 ± 13 | 109 ± 12 | NS |

Vales are means ± SE. *P* values were calculated for changes over time within each dose of leucine ingestion by one-way rmANOVA. NS stands for not significant.

**Table S3** Concentrations of plasma BCAAs, methionine, and AAAs in the isoleucine ingestion experiments

| Plasma amino acid | Ile ingested (mg/kg BW) | Time after ingestion (min) | | | | | | | | | | | P value |
| --- | --- | --- | --- | --- | --- | --- | --- | --- | --- | --- | --- | --- | --- |
|  |  | 0 | 15 | 30 | 45 | 60 | 75 | 90 | 105 | 120 | 180 | 240 |  |
| (μM) | | | | | | | | | | | | | |
| Leu | 0 | 125 ± 7 | 117 ± 6 | 112 ± 6 | 106 ± 7 | 105 ± 6 | 104 ± 6 | 106 ± 6 | 112 ± 6 | 106 ± 12 | 113 ± 3 | 118 ± 2 | NS |
|  | 10 | 134 ± 3 | 141 ± 4 | 129 ± 5 | 121 ± 4 | 118 ± 5 | 117 ± 5 | 116 ± 6 | 115 ± 5 | 115 ± 5 | 116 ± 5 | 123 ± 4 | <0.05 |
|  | 20 | 125 ± 4 | 129 ± 5 | 122 ± 5 | 116 ± 4 | 113 ± 3 | 112 ± 3 | 109 ± 3 | 113 ± 3 | 111 ± 3 | 113 ± 3 | 122 ± 3 | <0.05 |
|  | 30 | 122 ± 4 | 130 ± 5 | 123 ± 4 | 112 ± 3 | 107 ± 2 | 104 ± 3 | 106 ± 3 | 105 ± 2 | 105 ± 3 | 105 ± 4 | 109 ± 2 | <0.05 |
|  | 45 | 121 ± 7 | 125 ± 6 | 122 ± 7 | 110 ± 6 | 104 ± 4 | 100 ± 6 | 96 ± 5 | 95 ± 4 | 106 ± 11 | 100 ± 5 | 104 ± 6 | <0.05 |
|  | 60 | 122 ± 3 | 131 ± 3 | 127 ± 3 | 119 ± 4 | 107 ± 3 | 102 ± 4 | 99 ± 4 | 100 ± 3 | 100 ± 4 | 101 ± 3 | 105 ± 3 | <0.05 |
|  | 75 | 128 ± 7 | 133 ± 6 | 129 ± 8 | 117 ± 8 | 108 ± 8 | 100 ± 7 | 102 ± 6 | 106 ± 7 | 103 ± 6 | 106 ± 4 | 114 ± 4 | <0.05 |
|  | 90 | 116 ± 6 | 120 ± 5 | 119 ± 4 | 107 ± 6 | 97 ± 6 | 92 ± 6 | 88 ± 6 | 88 ± 6 | 88 ± 6 | 89 ± 5 | 92 ± 6 | <0.05 |
| Ile | 0 | 63 ± 4 | 58 ± 4 | 55 ± 4 | 52 ± 4 | 51 ± 3 | 50 ± 3 | 51 ± 3 | 48 ± 3 | 50 ± 3 | 52 ± 2 | 54 ± 2 | NS |
|  | 10 | 64 ± 2 | 163 ± 35 | 172 ± 19 | 156 ± 10 | 147 ± 7 | 137 ± 6 | 130 ± 5 | 123 ± 4 | 116 ± 4 | 102 ± 4 | 96 ± 4 | <0.05 |
|  | 20 | 61 ± 4 | 290 ± 68 | 308 ± 31 | 265 ± 18 | 240 ± 10 | 214 ± 10 | 193 ± 7 | 188 ± 7 | 174 ± 7 | 147 ± 6 | 135 ± 4 | <0.05 |
|  | 30 | 62 ± 2 | 412 ± 88 | 453 ± 49 | 374 ± 16 | 324 ± 16 | 297 ± 11 | 280 ± 10 | 261 ± 10 | 246 ± 9 | 202 ± 12 | 175 ± 7 | <0.05 |
|  | 45 | 63 ± 5 | 370 ± 88 | 562 ± 70 | 501 ± 41 | 440 ± 30 | 414 ± 26 | 373 ± 20 | 357 ± 16 | 337 ± 18 | 282 ± 15 | 241 ± 14 | <0.05 |
|  | 60 | 64 ± 2 | 491 ± 112 | 758 ± 108 | 744 ± 47 | 663 ± 34 | 574 ± 32 | 521 ± 17 | 494 ± 20 | 456 ± 19 | 367 ± 17 | 309 ± 10 | <0.05 |
|  | 75 | 70 ± 5 | 726 ± 145 | 946 ± 83 | 972 ± 101 | 825 ± 77 | 715 ± 47 | 664 ± 44 | 618 ± 35 | 576 ± 32 | 448 ± 31 | 370 ± 25 | <0.05 |
|  | 90 | 63 ± 4 | 545 ± 126 | 997 ± 138 | 1066 ± 78 | 999 ± 94 | 883 ± 59 | 803 ± 53 | 752 ± 42 | 697 ± 34 | 537 ± 30 | 426 ± 29 | <0.05 |
| Val | 0 | 248 ± 9 | 239 ± 9 | 236 ± 8 | 230 ± 8 | 226 ± 7 | 223 ± 8 | 225 ± 8 | 215 ± 8 | 223 ± 7 | 225 ± 7 | 230 ± 7 | NS |
|  | 10 | 260 ± 9 | 272 ± 11 | 259 ± 11 | 251 ± 9 | 246 ± 11 | 246 ± 11 | 243 ± 12 | 242 ± 10 | 238 ± 10 | 238 ± 11 | 240 ± 9 | NS |
|  | 20 | 242 ± 8 | 249 ± 9 | 246 ± 9 | 237 ± 9 | 234 ± 8 | 231 ± 8 | 224 ± 7 | 231 ± 9 | 224 ± 8 | 221 ± 7 | 226 ± 6 | NS |
|  | 30 | 240 ± 6 | 250 ± 8 | 244 ± 7 | 235 ± 6 | 228 ± 5 | 223 ± 6 | 225 ± 6 | 222 ± 5 | 222 ± 5 | 215 ± 10 | 214 ± 6 | NS |
|  | 45 | 238 ± 10 | 240 ± 10 | 245 ± 13 | 232 ± 13 | 225 ± 10 | 220 ± 12 | 215 ± 11 | 211 ± 8 | 212 ± 10 | 211 ± 9 | 211 ± 10 | NS |
|  | 60 | 240 ± 6 | 252 ± 6 | 249 ± 8 | 245 ± 8 | 231 ± 8 | 225 ± 8 | 218 ± 8 | 221 ± 7 | 215 ± 8 | 211 ± 7 | 210 ± 6 | <0.05 |
|  | 75 | 257 ± 16 | 262 ± 15 | 266 ± 18 | 254 ± 18 | 244 ± 17 | 231 ± 15 | 234 ± 15 | 234 ± 15 | 232 ± 15 | 225 ± 13 | 225 ± 12 | NS |
|  | 90 | 232 ± 7 | 239 ± 8 | 244 ± 7 | 233 ± 9 | 222 ± 10 | 217 ± 9 | 210 ± 10 | 209 ± 10 | 206 ± 9 | 199 ± 8 | 190 ± 11 | <0.05 |
| Met | 0 | 34 ± 1 | 33 ± 2 | 31 ± 2 | 30 ± 2 | 29 ± 2 | 28 ± 2 | 29 ± 2 | 27 ± 2 | 28 ± 2 | 28 ± 1 | 28 ± 1 | NS |
|  | 10 | 34 ± 1 | 35 ± 1 | 32 ± 2 | 31 ± 2 | 29 ± 2 | 30 ± 1 | 29 ± 1 | 29 ± 1 | 28 ± 1 | 27 ± 1 | 28 ± 1 | <0.05 |
|  | 20 | 35 ± 1 | 35 ± 1 | 33 ± 1 | 31 ± 1 | 31 ± 1 | 30 ± 1 | 29 ± 1 | 30 ± 1 | 27 ± 1 | 28 ± 1 | 28 ± 1 | <0.05 |
|  | 30 | 30 ± 1 | 31 ± 1 | 30 ± 1 | 28 ± 1 | 28 ± 1 | 26 ± 1 | 27 ± 1 | 26 ± 1 | 26 ± 1 | 25 ± 1 | 26 ± 1 | <0.05 |
|  | 45 | 31 ± 1 | 32 ± 2 | 31 ± 2 | 29 ± 1 | 29 ± 1 | 27 ± 2 | 26 ± 1 | 25 ± 1 | 25 ± 1 | 24 ± 1 | 25 ± 1 | <0.05 |
|  | 60 | 33 ± 1 | 34 ± 1 | 34 ± 1 | 32 ± 1 | 30 ± 1 | 29 ± 1 | 27 ± 1 | 28 ± 1 | 26 ± 1 | 26 ± 1 | 26 ± 1 | <0.05 |
|  | 75 | 32 ± 1 | 32 ± 1 | 32 ± 1 | 31 ± 1 | 30 ± 1 | 27 ± 1 | 27 ± 0 | 27 ± 1 | 27 ± 1 | 26 ± 1 | 28 ± 1 | <0.05 |
|  | 90 | 30 ± 1 | 31 ± 1 | 30 ± 1 | 29 ± 1 | 27 ± 1 | 27 ± 2 | 25 ± 1 | 26 ± 2 | 24 ± 1 | 23 ± 1 | 23 ± 1 | <0.05 |
| Phe | 0 | 68 ± 3 | 67 ± 3 | 63 ± 2 | 60 ± 3 | 60 ± 3 | 59 ± 2 | 58 ± 2 | 56 ± 2 | 58 ± 3 | 59 ± 1 | 62 ± 1 | NS |
|  | 10 | 72 ± 2 | 76 ± 2 | 70 ± 3 | 67 ± 2 | 65 ± 2 | 66 ± 2 | 65 ± 2 | 64 ± 2 | 64 ± 2 | 62 ± 3 | 66 ± 2 | <0.05 |
|  | 20 | 72 ± 2 | 73 ± 2 | 68 ± 2 | 66 ± 2 | 65 ± 1 | 64 ± 1 | 62 ± 1 | 63 ± 1 | 61 ± 2 | 62 ± 2 | 64 ± 2 | <0.05 |
|  | 30 | 65 ± 1 | 67 ± 2 | 66 ± 1 | 62 ± 1 | 61 ± 1 | 59 ± 2 | 60 ± 1 | 59 ± 1 | 59 ± 1 | 58 ± 1 | 59 ± 1 | <0.05 |
|  | 45 | 67 ± 2 | 67 ± 2 | 66 ± 2 | 62 ± 1 | 61 ± 1 | 59 ± 2 | 57 ± 1 | 56 ± 2 | 56 ± 1 | 57 ± 1 | 59 ± 2 | <0.05 |
|  | 60 | 65 ± 1 | 68 ± 2 | 67 ± 1 | 65 ± 1 | 60 ± 1 | 58 ± 1 | 57 ± 1 | 58 ± 1 | 56 ± 1 | 57 ± 2 | 59 ± 1 | <0.05 |
|  | 75 | 68 ± 1 | 68 ± 1 | 69 ± 1 | 64 ± 2 | 62 ± 1 | 59 ± 1 | 59 ± 1 | 60 ± 1 | 61 ± 1 | 59 ± 1 | 63 ± 1 | <0.05 |
|  | 90 | 62 ± 3 | 64 ± 3 | 64 ± 3 | 60 ± 3 | 57 ± 3 | 55 ± 2 | 54 ± 2 | 54 ± 3 | 55 ± 2 | 54 ± 2 | 55 ± 3 | NS |
| Trp | 0 | 59 ± 5 | 56 ± 5 | 55 ± 5 | 54 ± 5 | 52 ± | 551 ± 5 | 52 ± 4 | 49 ± 4 | 51 ± 3 | 48 ± 4 | 50 ± 3 | NS |
|  | 10 | 63 ± 3 | 62 ± 3 | 60 ± 3 | 57 ± 3 | 58 ± 3 | 57 ± 2 | 57 ± 2 | 57 ± 2 | 56 ± 3 | 52 ± 3 | 53 ± 2 | <0.05 |
|  | 20 | 66 ± 4 | 66 ± 4 | 64 ± 3 | 62 ± 3 | 59 ± 3 | 58 ± 3 | 56 ± 3 | 56 ± 3 | 56 ± 3 | 52 ± 3 | 54 ± 3 | <0.05 |
|  | 30 | 61 ± 4 | 61 ± 4 | 59 ± 3 | 57 ± 4 | 55 ± 4 | 53 ± 4 | 56 ± 4 | 53 ± 4 | 55 ± 4 | 51 ± 3 | 50 ± 3 | <0.05 |
|  | 45 | 62 ± 4 | 63 ± 4 | 59 ± 3 | 56 ± 3 | 55 ± 3 | 54 ± 3 | 54 ± 4 | 52 ± 4 | 52 ± 3 | 50 ± 3 | 50 ± 3 | NS |
|  | 60 | 61 ± 3 | 62 ± 3 | 59 ± 3 | 60 ± 3 | 56 ± 3 | 54 ± 3 | 54 ± 3 | 55 ± 3 | 53 ± 3 | 51 ± 3 | 50 ± 2 | NS |
|  | 75 | 64 ± 4 | 61 ± 3 | 61 ± 2 | 58 ± 3 | 56 ± 3 | 55 ± 4 | 54 ± 4 | 54 ± 4 | 53 ± 3 | 52 ± 3 | 52 ± 2 | NS |
|  | 90 | 60 ± 5 | 62 ± 5 | 60 ± 4 | 55 ± 3 | 54 ± 4 | 54 ± 5 | 53 ± 4 | 52 ± 5 | 51 ± 4 | 49 ± 4 | 48 ± 3 | NS |
| Tyr | 0 | 76 ± 4 | 72 ± 5 | 69 ± 5 | 67 ± 5 ± | 66 ± 5 | 64 ± 5 | 64 ± 4 | 61 ± 5 | 63 ± 4 | 63 ± 4 | 63 ± 3 | NS |
|  | 10 | 82 ± 4 | 84 ± 4 | 79 ± 6 | 76 ± 5 | 74 ± 5 | 74 ± 5 | 72 ± 5 | 71 ± 5 | 70 ± 4 | 67 ± 5 | 69 ± 4 | NS |
|  | 20 | 79 ± 6 | 80 ± 6 | 76 ± 5 | 74 ± 5 | 72 ± 5 | 70 ± 5 | 68 ± 5 | 70 ± 4 | 67 ± 6 | 67 ± 6 | 67 ± 5 | NS |
|  | 30 | 73 ± 4 | 75 ± 5 | 73 ± 4 | 69 ± 5 | 68 ± 4 | 65 ± 5 | 66 ± 4 | 64 ± 5 | 65 ± 4 | 64 ± 5 | 62 ± 6 | NS |
|  | 45 | 73 ± 5 | 73 ± 4 | 72 ± 5 | 68 ± 5 | 66 ± 5 | 64 ± 6 | 63 ± 6 | 60 ± 6 | 61 ± 5 | 60 ± 5 | 60 ± 5 | NS |
|  | 60 | 76 ± 5 | 79 ± 4 | 76 ± 5 | 75 ± 4 | 70 ± 5 | 69 ± 5 | 67 ± 5 | 66 ± 5 | 66 ± 5 | 64 ± 5 | 63 ± 5 | NS |
|  | 75 | 77 ± 5 | 77 ± 5 | 75 ± 5 | 72 ± 6 | 70 ± 5 | 66 ± 5 | 66 ± 5 | 66 ± 5 | 67 ± 5 | 64 ± 5 | 65 ± 4 | NS |
|  | 90 | 71 ± 4 | 73 ± 3 | 71 ± 3 | 67 ± 3 | 64 ± 3 | 63 ± 3 | 60 ± 3 | 60 ± 3 | 60 ± 3 | 57 ± 3 | 55 ± 4 | <0.05 |

Values are means ± SE. *P* values were calculated for changes over time within each dose of leucine ingestion by one-way rmANOVA. NS stands for not significant.

**Table S4** Concentrations of plasma amino acids other than BCAAs, methionine, and AAAs in the isoleucine ingestion experiments

| Plasma amino acid | Ile ingested (mg/kg BW) | Time after ingestion (min) | | | | | | | | | | | P value |
| --- | --- | --- | --- | --- | --- | --- | --- | --- | --- | --- | --- | --- | --- |
|  |  | 0 | 15 | 30 | 45 | 60 | 75 | 90 | 105 | 120 | 180 | 240 |  |
| (μM) | | | | | | | | | | | | | |
| Ala | 0 | 375 ± 55 | 392 ± 54 | 389 ± 61 | 378 ± 53 | 374 ± 60 | 361 ± 69 | 368 ± 48 | 343 ± 73 | 346 ± 53 | 352 ± 50 | 336 ± 71 | NS |
|  | 10 | 422 ± 61 | 444 ± 49 | 415 ± 67 | 404 ± 68 | 387 ± 64 | 396 ± 69 | 386 ± 65 | 386 ± 63 | 371 ± 60 | 358 ± 72 | 363 ± 65 | NS |
|  | 20 | 395 ± 49 | 393 ± 46 | 378 ± 43 | 355 ± 46 | 356 ± 33 | 344 ± 39 | 328 ± 41 | 331 ± 33 | 308 ± 39 | 302 ± 33 | 288 ± 40 | <0.05 |
|  | 30 | 380 ± 35 | 386 ± 32 | 378 ± 30 | 370 ± 33 | 365 ± 36 | 341 ± 45 | 345 ± 26 | 332 ± 38 | 335 ± 38 | 325 ± 48 | 314 ± 48 | NS |
|  | 45 | 407 ± 60 | 411 ± 57 | 405 ± 55 | 397 ± 53 | 395 ± 50 | 379 ± 67 | 383 ± 59 | 372 ± 58 | 370 ± 48 | 360 ± 51 | 365 ± 48 | NS |
|  | 60 | 422 ± 64 | 438 ± 56 | 424 ± 60 | 429 ± 51 | 404 ± 57 | 394 ± 51 | 391 ± 58 | 392 ± 50 | 378 ± 56 | 378 ± 62 | 368 ± 48 | NS |
|  | 75 | 417 ± 53 | 403 ± 58 | 408 ± 52 | 398 ± 61 | 393 ± 54 | 373 ± 54 | 386 ± 47 | 373 ± 54 | 385 ± 51 | 369 ± 57 | 377 ± 60 | NS |
|  | 90 | 408 ± 58 | 419 ± 54 | 418 ± 52 | 411 ± 55 | 401 ± 56 | 404 ± 50 | 399 ± 57 | 390 ± 58 | 398 ± 60 | 391 ± 64 | 376 ± 75 | NS |
| Arg | 0 | 117 ± 16 | 115 ± 19 | 109 ± 16 | 108 ± 17 | 106 ± 17 | 103 ± 17 | 104 ± 14 | 99 ± 18 | 101 ± 17 | 101 ± 16 | 103 ± 10 | NS |
|  | 10 | 113 ± 16 | 122 ± 14 | 113 ± 16 | 110 ± 16 | 107 ± 13 | 108 ± 15 | 107 ± 13 | 106 ± 13 | 104 ± 13 | 99 ± 14 | 100 ± 16 | NS |
|  | 20 | 112 ± 12 | 115 ± 13 | 109 ± 11 | 105 ± 8 | 103 ± 9 | 103 ± 9 | 97 ± 8 | 100 ± 9 | 96 ± 7 | 97 ± 15 | 95 ± 8 | NS |
|  | 30 | 105 ± 12 | 110 ± 16 | 105 ± 12 | 102 ± 11 | 100 ± 11 | 96 ± 12 | 99 ± 12 | 96 ± 10 | 95 ± 10 | 92 ± 6 | 92 ± 9 | NS |
|  | 45 | 106 ± 14 | 109 ± 12 | 110 ± 14 | 105 ± 11 | 102 ± 10 | 101 ± 14 | 97 ± 13 | 95 ± 10 | 95 ± 8 | 94 ± 7 | 94 ± 10 | NS |
|  | 60 | 111 ± 10 | 117 ± 11 | 113 ± 10 | 114 ± 10 | 107 ± 9 | 105 ± 9 | 104 ± 10 | 105 ± 10 | 103 ± 9 | 100 ± 14 | 99 ± 10 | NS |
|  | 75 | 105 ± 6 | 105 ± 7 | 108 ± 7 | 103 ± 7 | 101 ± 8 | 98 ± 7 | 98 ± 8 | 96 ± 8 | 97 ± 8 | 94 ± 8 | 98 ± 8 | NS |
|  | 90 | 101 ± 12 | 104 ± 11 | 106 ± 10 | 102 ± 8 | 98 ± 9 | 98 ± 8 | 96 ± 7 | 96 ± 10 | 95 ± 9 | 91 ± 10 | 89 ± 10 | NS |
| Asn | 0 | 54 ± 8 | 52 ± 9 | 53 ± 9 | 51 ± 9 | 51 ± 8 | 49 ± 9 | 50 ± 8 | 47 ± 10 | 47 ± 7 | 49 ± 7 | 49 ± 7 | NS |
|  | 10 | 51 ± 8 | 55 ± 8 | 51 ± 10 | 50 ± 8 | 48 ± 7 | 49 ± 6 | 48 ± 7 | 48 ± 6 | 47 ± 5 | 47 ± 7 | 47 ± 7 | NS |
|  | 20 | 53 ± 7 | 52 ± 7 | 51 ± 5 | 48 ± 6 | 47 ± 5 | 47 ± 5 | 45 ± 6 | 46 ± 4 | 45 ± 6 | 44 ± 7 | 45 ± 6 | NS |
|  | 30 | 48 ± 5 | 51 ± 5 | 49 ± 5 | 47 ± 5 | 47 ± 5 | 44 ± 6 | 45 ± 5 | 45 ± 5 | 45 ± 5 | 43 ± 5 | 44 ± 4 | NS |
|  | 45 | 48 ± 5 | 49 ± 4 | 49 ± 4 | 46 ± 5 | 47 ± 5 | 45 ± 6 | 44 ± 6 | 44 ± 6 | 44 ± 4 | 44 ± 5 | 45 ± 5 | NS |
|  | 60 | 51 ± 6 | 52 ± 5 | 52 ± 5 | 52 ± 3 | 48 ± 5 | 47 ± 4 | 46 ± 4 | 47 ± 4 | 46 ± 5 | 46 ± 5 | 46 ± 3 | NS |
|  | 75 | 47 ± 4 | 47 ± 5 | 47 ± 4 | 45 ± 4 | 44 ± 4 | 42 ± 5 | 42 ± 4 | 43 ± 4 | 44 ± 4 | 43 ± 5 | 44 ± 3 | NS |
|  | 90 | 45 ± 4 | 45 ± 3 | 45 ± 3 | 44 ± 3 | 42 ± 4 | 43 ± 3 | 41 ± 3 | 42 ± 3 | 42 ± 3 | 40 ± 4 | 40 ± 5 | NS |
| Asp | 0 | 0.3 ± 0.6 | 0.5 ± 0.6 | 0.3 ± 1.0 | 0.4 ± 0.6 | 0.3 ± 0.9 | 0.3 ± 0.6 | 0.3 ± 0.7 | 0.4 ± 0.6 | 0.9 ± 1.3 | 0.4 ± 0.7 | 0.4 ± 0.8 | NS |
|  | 10 | 2.0 ± 0.3 | 2.1 ± 0.4 | 2.2 ± 0.3 | 1.7 ± 0.9 | 2.2 ± 0.4 | 2.0 ± 0.3 | 1.7±1.0 | 1.9 ± 0.3 | 1.9 ± 0.4 | 2.0 ± 0.4 | 2.0±0.7 | NS |
|  | 20 | 1.7 ± 1.0 | 2.0 ± 0.4 | 1.9 ± 0.4 | 1.7 ± 1.0 | 2.0 ± 0.3 | 2.0 ± 0.3 | 1.6 ± 0.8 | 1.8 ± 0.9 | 2.2 ± 0.3 | 1.9 ± 0.3 | 1.3 ± 1.0 | NS |
|  | 30 | 2.2 ± 0.5 | 2.2 ± 0.4 | 1.7 ± 0.9 | 1.8 ± 0.3 | 1.4 ± 0.8 | 2.1 ± 0.2 | 1.6 ± 0.8 | 1.9 ± 0.2 | 1.8 ± 0.9 | 1.7 ± 0.9 | 1.5 ± 0.8 | NS |
|  | 45 | 1.5 ± 0.8 | 1.1 ± 0.8 | 1.8 ± 0.7 | 0.7 ± 0.9 | 0.7 ± 0.8 | 1.0 ± 0.9 | 1.3 ± 0.7 | 1.1 ± 0.8 | 0.8 ± 1.0 | 0.8 ± 0.9 | 0.9 ± 0.7 | NS |
|  | 60 | 1.5 ± 0.8 | 1.3 ± 0.9 | 1.0 ± 1.3 | 1.1 ± 0.9 | 1.2 ± 1.0 | 1.2 ± 1.0 | 1.5 ± 1.0 | 2.1 ± 0.4 | 1.3 ± 0.8 | 1.0 ± 0.9 | 1.4 ± 0.9 | NS |
|  | 75 | 2.0 ± 0.5 | 2.1 ± 0.5 | 2.0 ± 0.3 | 1.8 ± 0.2 | 1.9 ± 0.2 | 2.0 ± 0.3 | 1.8 ± 0.2 | 2.5 ± 0.4 | 2.5 ± 0.3 | 2.3 ± 0.3 | 2.2 ± 0.5 | <0.05 |
|  | 90 | 1.8 ± 0.3 | 1.8 ± 0.3 | 1.8 ± 0.2 | 1.8 ± 0.1 | 1.7 ± 0.3 | 1.9 ± 0.2 | 1.8 ± 0.2 | 2.2 ± 0.3 | 2.0 ± 0.2 | 2.2 ± 0.4 | 1.8 ± 0.3 | NS |
| Cys | 0 | 51 ± 3 | 49 ± 4 | 50 ± 5 | 50 ± 4 | 49 ± 4 | 49 ± 4 | 49 ± 5 | 49 ± 5 | 50 ± 6 | 48 ± 4 | 48 ± 3 | NS |
|  | 10 | 57 ± 4 | 58 ± 6 | 56 ± 6 | 55 ± 6 | 56 ± 5 | 55 ± 5 | 55 ± 5 | 54 ± 4 | 54 ± 4 | 54 ± 5 | 53 ± 4 | NS |
|  | 20 | 55 ± 5 | 54 ± 4 | 55 ± 5 | 55 ± 5 | 56 ± 4 | 54 ± 5 | 54 ± 5 | 56 ± 6 | 54 ± 6 | 53 ± 5 | 52 ± 4 | NS |
|  | 30 | 55 ± 6 | 55 ± 6 | 53 ± 6 | 53 ± 5 | 52 ± 5 | 52 ± 4 | 52 ± 6 | 52 ± 5 | 53 ± 5 | 51 ± 6 | 50 ± 5 | NS |
|  | 45 | 51 ± 4 | 50 ± 5 | 52 ± 5 | 50 ± 5 | 49 ± 5 | 49 ± 5 | 49 ± 4 | 49 ± 4 | 50 ± 6 | 50 ± 5 | 49 ± 6 | NS |
|  | 60 | 54 ± 7 | 54 ± 7 | 53 ± 7 | 52 ± 6 | 51 ± 6 | 52 ± 7 | 50 ± 7 | 51 ± 5 | 50 ± 7 | 52 ± 6 | 50 ± 5 | NS |
|  | 75 | 57 ± 7 | 57 ± 8 | 57 ± 6 | 56 ± 6 | 56 ± 6 | 54 ± 5 | 57 ± 6 | 57 ± 7 | 56 ± 6 | 57 ± 7 | 55 ± 5 | NS |
|  | 90 | 50 ± 6 | 50 ± 6 | 51 ± 3 | 50 ± 4 | 50 ± 5 | 51 ± 7 | 49 ± 4 | 51 ± 6 | 50 ± 4 | 51 ± 3 | 48 ± 6 | NS |
| Gln | 0 | 624 ± 40 | 621 ± 63 | 614 ± 59 | 600 ± 61 | 602 ± 70 | 588 ± 69 | 592 ± 61 | 552 ± 89 | 582 ± 75 | 620 ± 55 | 630 ± 32 | NS |
|  | 10 | 604 ± 54 | 641 ± 57 | 606 ± 74 | 603 ± 77 | 573 ± 41 | 592 ± 32 | 598 ± 47 | 602 ± 42 | 589 ± 50 | 608 ± 61 | 639 ± 48 | NS |
|  | 20 | 634 ± 51 | 628 ± 55 | 606 ± 37 | 584 ± 49 | 567 ± 37 | 588 ± 47 | 560 ± 43 | 584 ± 41 | 562 ± 14 | 594 ± 26 | 625 ± 28 | NS |
|  | 30 | 559 ± 40 | 551 ± 22 | 563 ± 39 | 555 ± 29 | 547 ± 26 | 523 ± 29 | 548 ± 42 | 540 ± 28 | 538 ± 23 | 559 ± 29 | 583 ± 15 | <0.05 |
|  | 45 | 627 ± 24 | 633 ± 24 | 634 ± 24 | 616 ± 30 | 624 ± 29 | 609 ± 53 | 602 ± 49 | 588 ± 55 | 596 ± 37 | 611 ± 24 | 633 ± 30 | NS |
|  | 60 | 606 ± 53 | 620 ± 47 | 617 ± 65 | 632 ± 34 | 597 ± 42 | 586 ± 40 | 574 ± 63 | 596 ± 25 | 584 ± 42 | 588 ± 47 | 623 ± 33 | NS |
|  | 75 | 580 ± 31 | 569 ± 29 | 595 ± 24 | 576 ± 20 | 571 ± 24 | 550 ± 34 | 562 ± 16 | 561 ± 13 | 579 ± 19 | 584 ± 21 | 623 ± 34 | <0.05 |
|  | 90 | 590 ± 50 | 607 ± 35 | 612 ± 47 | 595 ± 33 | 577 ± 39 | 586 ± 52 | 572 ± 39 | 578 ± 47 | 591 ± 35 | 586 ± 25 | 592 ± 38 | NS |
| Glu | 0 | 26 ± 7 | 27 ± 12 | 33 ± 11 | 35 ± 8 | 40 ± 15 | 38 ± 15 | 38 ± 12 | 41 ± 13 | 42 ± 13 | 34 ± 10 | 34 ± 10 | NS |
|  | 10 | 46 ± 8 | 44 ± 15 | 48 ± 14 | 45 ± 17 | 50 ± 7 | 44 ± 11 | 42 ± 10 | 40 ± 9 | 43 ± 7 | 45 ± 12 | 34 ± 8 | NS |
|  | 20 | 39 ± 16 | 40 ± 18 | 45 ± 13 | 47 ± 13 | 46 ± 10 | 44 ± 13 | 47 ± 12 | 47 ± 10 | 51 ± 11 | 45 ± 15 | 39 ± 13 | NS |
|  | 30 | 48 ± 13 | 49 ± 12 | 44 ± 11 | 43 ± 6 | 39 ± 11 | 43 ± 4 | 39 ± 11 | 45 ± 7 | 44 ± 10 | 40 ± 11 | 37 ± 11 | NS |
|  | 45 | 38 ± 10 | 35 ± 11 | 44 ± 10 | 40 ± 11 | 35 ± 9 | 40 ± 9 | 38 ± 10 | 40 ± 12 | 41 ± 8 | 37 ± 10 | 34 ± 12 | NS |
|  | 60 | 44 ± 7 | 42 ± 12 | 41 ± 11 | 41 ± 7 | 41 ± 6 | 43 ± 9 | 38 ± 6 | 41 ± 9 | 43 ± 8 | 39 ± 6 | 34 ± 7 | NS |
|  | 75 | 45 ± 15 | 52 ± 15 | 45 ± 10 | 44 ± 9 | 41 ± 8 | 42 ± 4 | 44 ± 8 | 50 ± 8 | 44 ± 6 | 48 ± 9 | 40 ± 11 | NS |
|  | 90 | 37 ± 4 | 35 ± 13 | 40 ± 8 | 38 ± 8 | 38 ± 12 | 37 ± 9 | 37 ± 10 | 44 ± 8 | 41 ± 10 | 42 ± 10 | 37 ± 10 | NS |
| (Table S4-2) | | | | | | | | | | | | | |
| (μM) | | | | | | | | | | | | | |
| Gly | 0 | 266 ± 16 | 266 ± 19 | 264 ± 11 | 259 ± 16 | 258 ± 19 | 254 ± 15 | 257 ± 21 | 242 ± 20 | 252 ± 28 | 255 ± 17 | 255 ± 9 | NS |
|  | 10 | 264 ± 18 | 279 ± 15 | 267 ± 14 | 264 ± 17 | 258 ± 14 | 264 ± 18 | 262 ± 82 | 62 ± 12 | 256 ± 92 | 54 ± 11 | 262 ± 10 | NS |
|  | 20 | 254 ± 20 | 254 ± 21 | 246 ± 21 | 243 ± 20 | 239 ± 22 | 242 ± 23 | 232 ± 21 | 239 ± 26 | 229 ± 18 | 226 ± 15 | 230 ± 18 | NS |
|  | 30 | 242 ± 13 | 239 ± 15 | 238 ± 15 | 236 ± 15 | 235 ± 14 | 227 ± 16 | 235 ± 14 | 230 ± 14 | 228 ± 11 | 227 ± 19 | 225 ± 14 | NS |
|  | 45 | 240 ± 25 | 239 ± 27 | 236 ± 25 | 229 ± 22 | 231 ± 28 | 226 ± 25 | 223 ± 20 | 220 ± 19 | 220 ± 17 | 219 ± 19 | 225 ± 24 | NS |
|  | 60 | 260 ± 18 | 267 ± 25 | 258 ± 28 | 260 ± 21 | 248 ± 13 | 245 ± 14 | 239 ± 18 | 247 ± 15 | 241 ± 13 | 237 ± 22 | 41 ± 19 | NS |
|  | 75 | 240 ± 23 | 233 ± 23 | 233 ± 23 | 224 ± 19 | 224 ± 20 | 217 ± 16 | 220 ± 16 | 223 ± 18 | 225 ± 16 | 221 ± 18 | 229 ± 22 | NS |
|  | 90 | 248 ± 25 | 250 ± 29 | 247 ± 29 | 238 ± 24 | 232 ± 21 | 239 ± 24 | 235 ± 26 | 239 ± 19 | 240 ± 27 | 233 ± 19 | 227 ± 21 | NS |
| His | 0 | 90 ± 3 | 88 ± 4 | 88 ± 2 | 85 ± 3 | 86 ± 3 | 85 ± 3 | 87 ± 3 | 82 ± 4 | 85 ± 4 | 88 ± 3 | 88 ± 1 | NS |
|  | 10 | 93 ± 2 | 96 ± 4 | 93 ± 2 | 91 ± 2 | 89 ± 1 | 91 ± 1 | 91 ± 1 | 91 ± 1 | 90 ± 1 | 91 ± 1 | 93 ± 2 | NS |
|  | 20 | 92 ± 1 | 93 ± 1 | 93 ± 1 | 89 ± 1 | 88 ± 2 | 89 ± 1 | 86 ± 1 | 89 ± 2 | 86 ± 2 | 86 ± 1 | 87 ± 1 | <0.05 |
|  | 30 | 84 ± 3 | 85 ± 3 | 87 ± 3 | 85 ± 1 | 85 ± 2 | 81 ± 2 | 83 ± 2 | 82 ± 2 | 83 ± 2 | 82 ± 1 | 83 ± 2 | <0.05 |
|  | 45 | 84 ± 4 | 85 ± 4 | 87 ± 5 | 84 ± 4 | 83 ± 2 | 81 ± 3 | 81 ± 3 | 79 ± 3 | 80 ± 4 | 81 ± 3 | 83 ± 4 | NS |
|  | 60 | 91 ± 3 | 94 ± 3 | 93 ± 2 | 94 ± 3 | 91 ± 3 | 88 ± 3 | 88 ± 2 | 89 ± 2 | 87 ± 2 | 88 ± 3 | 87 ± 2 | NS |
|  | 75 | 88 ± 2 | 90 ± 2 | 93 ± 2 | 90 ± 3 | 88 ± 2 | 85 ± 3 | 86 ± 2 | 87 ± 2 | 87 ± 2 | 88 ± 2 | 89 ± 2 | NS |
|  | 90 | 84 ± 5 | 89 ± 4 | 91 ± 5 | 89 ± 3 | 86 ± 3 | 86 ± 3 | 84 ± 3 | 85 ± 5 | 85 ± 4 | 83 ± 3 | 81 ± 4 | NS |
| Lys | 0 | 220 ± 17 | 219 ± 25 | 216 ± 23 | 208 ± 23 | 208 ± 24 | 204 ± 24 | 207 ± 19 | 196 ± 26 | 202 ± 23 | 204 ± 21 | 206 ± 14 | NS |
|  | 10 | 222 ± 23 | 236 ± 19 | 221 ± 26 | 216 ± 24 | 213 ± 16 | 219 ± 16 | 216 ± 18 | 216±15 | 213 ± 19 | 207 ± 21 | 212 ± 21 | NS |
|  | 20 | 221 ± 20 | 221 ± 22 | 215 ± 15 | 209 ± 14 | 208 ± 13 | 206 ± 15 | 199 ± 13 | 205 ± 14 | 197 ± 17 | 192 ± 16 | 195 ± 13 | <0.05 |
|  | 30 | 199 ± 18 | 202 ± 21 | 200 ± 19 | 194 ± 16 | 193 ± 16 | 187 ± 18 | 193 ± 18 | 189 ± 16 | 190 ± 17 | 184 ± 12 | 186 ± 14 | <0.05 |
|  | 45 | 206 ± 24 | 206 ± 23 | 203 ± 25 | 196 ± 21 | 196 ± 17 | 192 ± 26 | 190 ± 24 | 188 ± 23 | 186 ± 21 | 187 ± 21 | 188 ± 24 | NS |
|  | 60 | 206 ± 11 | 211 ± 12 | 207 ± 14 | 207 ± 7 | 197 ± 10 | 195 ± 7 | 193 ± 13 | 195 ± 7 | 191 ± 8 | 189 ± 15 | 190 ± 10 | <0.05 |
|  | 75 | 203 ± 13 | 198 ± 16 | 200 ± 11 | 193 ± 16 | 190 ± 13 | 184 ± 16 | 187 ± 12 | 187 ± 14 | 189 ± 11 | 184 ± 15 | 188 ± 9 | NS |
|  | 90 | 192 ± 26 | 196 ± 24 | 195 ± 24 | 187 ± 21 | 183 ± 24 | 183 ± 24 | 182 ± 21 | 182 ± 27 | 182 ± 23 | 177 ± 24 | 173 ± 26 | NS |
| Pro | 0 | 147 ± 17 | 147 ± 17 | 144 ± 12 | 145 ± 16 | 141 ± 15 | 142 ± 15 | 145 ± 20 | 137 ± 21 | 137 ± 17 | 134 ± 16 | 130 ± 14 | NS |
|  | 10 | 165 ± 25 | 170 ± 22 | 156 ± 24 | 157 ± 24 | 154 ± 29 | 152 ± 30 | 147 ± 28 | 147 ± 26 | 147 ± 26 | 142 ± 30 | 144 ± 26 | NS |
|  | 20 | 161 ± 11 | 156 ± 15 | 150 ± 12 | 145 ± 12 | 140 ± 16 | 138 ± 13 | 136 ± 17 | 136 ± 21 | 135 ± 15 | 130 ± 12 | 128 ± 14 | <0.05 |
|  | 30 | 157 ± 16 | 158 ± 20 | 151 ± 18 | 150 ± 18 | 145 ± 18 | 141 ± 17 | 140 ± 17 | 144 ± 17 | 140 ± 14 | 138 ± 21 | 139 ± 22 | NS |
|  | 45 | 172 ± 26 | 167 ± 25 | 160 ± 24 | 157 ± 23 | 154 ± 20 | 151 ± 30 | 148 ± 22 | 143 ± 21 | 144 ± 22 | 146 ± 22 | 146 ± 27 | NS |
|  | 60 | 155 ± 12 | 161 ± 13 | 154 ± 11 | 155 ± 17 | 139 ± 14 | 142 ± 14 | 141 ± 13 | 141 ± 15 | 139 ± 13 | 136 ± 15 | 132 ± 13 | NS |
|  | 75 | 161 ± 23 | 156 ± 22 | 152 ± 26 | 146 ± 27 | 143 ± 24 | 136 ± 21 | 141 ± 26 | 141 ± 23 | 144 ± 25 | 135 ± 22 | 136 ± 21 | NS |
|  | 90 | 157 ± 17 | 157 ± 17 | 153 ± 20 | 149 ± 16 | 141 ± 20 | 148 ± 15 | 141 ± 18 | 144 ± 20 | 144 ± 18 | 141 ± 20 | 136 ± 22 | NS |
| Ser | 0 | 129 ± 14 | 129 ± 13 | 129 ± 14 | 126 ± 14 | 125 ± 12 | 124 ± 11 | 126 ± 15 | 121 ± 13 | 125 ± 16 | 125 ± 12 | 126 ± 12 | NS |
|  | 10 | 128 ± 12 | 131 ± 7 | 127 ± 10 | 124 ± 8 | 124 ± 8 | 124 ± 7 | 124 ± 8 | 122 ± 9 | 121 ± 8 | 121 ± 7 | 130 ± 9 | NS |
|  | 20 | 129 ± 14 | 131 ± 15 | 127 ± 16 | 125 ± 16 | 126 ± 14 | 123 ± 13 | 122 ± 13 | 127 ± 14 | 124 ± 10 | 123 ± 10 | 124 ± 13 | NS |
|  | 30 | 128 ± 14 | 132 ± 10 | 129 ± 12 | 126 ± 13 | 123 ± 12 | 122 ± 12 | 124 ± 13 | 123 ± 13 | 125 ± 11 | 124 ± 13 | 122 ± 14 | NS |
|  | 45 | 123 ± 23 | 123 ± 23 | 125 ± 22 | 119 ± 21 | 117 ± 23 | 117 ± 18 | 115 ± 20 | 116 ± 18 | 117 ± 17 | 117 ± 19 | 120 ± 18 | NS |
|  | 60 | 126 ± 6 | 131 ± 7 | 127 ± 9 | 128 ± 8 | 122 ± 8 | 121 ± 7 | 119 ± 9 | 122 ± 7 | 121 ± 8 | 122 ± 9 | 121 ± 10 | NS |
|  | 75 | 126 ± 19 | 128 ± 17 | 127 ± 19 | 123 ± 16 | 122 ± 16 | 119 ± 18 | 123 ± 15 | 125 ± 16 | 125 ± 18 | 130 ± 16 | 127 ± 16 | NS |
|  | 90 | 123 ± 21 | 126 ± 17 | 128 ± 25 | 121 ± 19 | 119 ± 13 | 122 ± 17 | 120 ± 18 | 123 ± 16 | 123 ± 17 | 124 ± 19 | 121 ± 14 | NS |
| Thr | 0 | 160 ± 19 | 159 ± 21 | 157 ± 21 | 151 ± 20 | 150 ± 21 | 147 ± 22 | 149 ± 20 | 142 ± 23 | 146 ± 21 | 149 ± 19 | 149 ± 17 | NS |
|  | 10 | 158 ± 20 | 165 ± 16 | 156 ± 23 | 152 ± 21 | 148 ± 18 | 152 ± 19 | 150 ± 19 | 150 ± 16 | 147 ± 17 | 146 ± 21 | 150 ± 18 | NS |
|  | 20 | 153 ± 21 | 153 ± 20 | 148 ± 18 | 143 ± 16 | 142 ± 15 | 140 ± 18 | 137 ± 18 | 140 ± 17 | 135 ± 17 | 133 ± 17 | 135 ± 15 | NS |
|  | 30 | 143 ± 16 | 147 ± 19 | 144 ± 16 | 140 ± 17 | 138 ± 16 | 133 ± 19 | 137 ± 16 | 134 ± 17 | 135 ± 18 | 133 ± 18 | 133 ± 19 | NS |
|  | 45 | 151 ± 30 | 151 ± 28 | 149 ± 29 | 144 ± 30 | 143 ± 29 | 140 ± 33 | 139 ± 31 | 136 ± 30 | 137 ± 27 | 137 ± 29 | 139 ± 30 | NS |
|  | 60 | 163 ± 20 | 169 ± 17 | 164 ± 21 | 165 ± 15 | 156 ± 19 | 153 ± 16 | 151 ± 20 | 154 ± 16 | 151 ± 19 | 148 ± 21 | 149 ± 16 | NS |
|  | 75 | 142 ± 20 | 140 ± 20 | 142 ± 19 | 136 ± 22 | 134 ± 21 | 129 ± 21 | 131 ± 19 | 131 ± 21 | 133 ± 19 | 130 ± 22 | 133 ± 18 | NS |
|  | 90 | 138 ± 18 | 142 ± 16 | 141 ± 16 | 136 ± 18 | 131 ± 18 | 132 ± 17 | 130 ± 17 | 130 ± 15 | 131 ± 16 | 128 ± 18 | 126 ± 20 | NS |

Values are means ± SE. *P* values were calculated for changes over time within each dose of leucine ingestion by one-way rmANOVA. NS stands for not significant.

**Table S5** Concentrations of plasma BCAAs, methionine, and AAAs in the valine ingestion experiments

| Plasma amino acid | Val ingested (mg/kg BW) | Time after ingestion (min) | | | | | | | | | | | P value |
| --- | --- | --- | --- | --- | --- | --- | --- | --- | --- | --- | --- | --- | --- |
|  |  | 0 | 15 | 30 | 45 | 60 | 75 | 90 | 105 | 120 | 180 | 240 |  |
| (μM) | | | | | | | | | | | | | |
| Leu | 0 | 125 ± 7 | 117 ± 6 | 112 ± 6 | 106 ± 7 | 105 ± 6 | 104 ± 6 | 106 ± 6 | 112 ± 6 | 106 ± 12 | 113 ± 3 | 118 ± 2 | NS |
|  | 10 | 132 ± 6 | 129 ± 5 | 118 ± 6 | 119 ± 7 | 116 ± 7 | 114 ± 6 | 115 ± 8 | 114 ± 6 | 116 ± 5 | 114 ± 5 | 114 ± 5 | NS |
|  | 20 | 133 ± 5 | 138 ± 4 | 131 ± 5 | 122 ± 5 | 118 ± 5 | 113 ± 6 | 118 ± 5 | 117 ± 5 | 116 ± 5 | 119 ± 5 | 126 ± 2 | <0.05 |
|  | 30 | 126 ± 6 | 128 ± 5 | 122 ± 5 | 115 ± 7 | 112 ± 5 | 112 ± 6 | 111 ± 6 | 111 ± 7 | 114 ± 5 | 119 ± 5 | 122 ± 7 | NS |
|  | 45 | 128 ± 6 | 130 ± 6 | 120 ± 6 | 111 ± 3 | 107 ± 3 | 110 ± 4 | 104 ± 3 | 107 ± 3 | 110 ± 3 | 111 ± 4 | 115 ± 3 | <0.05 |
|  | 60 | 122 ± 2 | 130 ± 3 | 134 ± 5 | 127 ± 5 | 119 ± 4 | 118 ± 4 | 116 ± 4 | 116 ± 5 | 118 ± 4 | 121 ± 4 | 122 ± 3 | NS |
|  | 75 | 119 ± 5 | 127 ± 5 | 124 ± 4 | 116 ± 4 | 108 ± 4 | 106 ± 3 | 103 ± 4 | 103 ± 4 | 104 ± 4 | 105 ± 5 | 110 ± 2 | <0.05 |
|  | 90 | 117 ± 5 | 125 ± 6 | 124 ± 7 | 121 ± 8 | 115 ± 8 | 114 ± 3 | 112 ± 5 | 109 ± 5 | 107 ± 4 | 112 ± 3 | 110 ± 4 | NS |
| Ile | 0 | 63 ± 4 | 58 ± 4 | 55 ± 4 | 52 ± 4 | 51 ± 3 | 50 ± 3 | 51 ± 3 | 48 ± 3 | 50 ± 3 | 52 ± 2 | 54 ± 2 | NS |
|  | 10 | 66 ± 4 | 66 ± 4 | 60 ± 4 | 60 ± 5 | 57 ± 4 | 56 ± 4 | 57 ± 4 | 56 ± 4 | 57 ± 2 | 54 ± 3 | 53 ± 2 | NS |
|  | 20 | 68 ± 3 | 70 ± 3 | 67 ± 3 | 62 ± 3 | 59 ± 3 | 56 ± 4 | 58 ± 4 | 57 ± 3 | 56 ± 4 | 55 ± 3 | 63 ± 6 | NS |
|  | 30 | 67 ± 4 | 68 ± 3 | 65 ± 3 | 60 ± 4 | 59 ± 3 | 57 ± 3 | 57 ± 3 | 56 ± 4 | 58 ± 3 | 59 ± 3 | 58 ± 4 | NS |
|  | 45 | 71 ± 7 | 72 ± 7 | 65 ± 5 | 61 ± 5 | 58 ± 5 | 59 ± 5 | 56 ± 3 | 57 ± 5 | 58 ± 4 | 57 ± 2 | 58 ± 2 | NS |
|  | 60 | 68 ± 2 | 72 ± 2 | 75 ± 4 | 70 ± 3 | 65 ± 3 | 64 ± 3 | 62 ± 2 | 62 ± 3 | 63 ± 3 | 62 ± 2 | 60 ± 1 | <0.05 |
|  | 75 | 65 ± 3 | 70 ± 3 | 69 ± 3 | 64 ± 3 | 59 ± 2 | 57 ± 3 | 56 ± 2 | 55 ± 2 | 55 ± 2 | 54 ± 3 | 54 ± 2 | <0.05 |
|  | 90 | 61 ± 5 | 66 ± 5 | 65 ± 6 | 64 ± 7 | 60 ± 6 | 59 ± 4 | 57 ± 4 | 55 ± 4 | 53 ± 3 | 54 ± 2 | 53 ± 2 | NS |
| Val | 0 | 248 ± 9 | 239 ± 9 | 236 ± 8 | 230 ± 8 | 226 ± 7 | 223 ± 8 | 225 ± 8 | 215 ± 8 | 223 ± 7 | 225 ± 7 | 230 ± 7 | NS |
|  | 10 | 266 ± 10 | 377 ± 38 | 387 ± 30 | 387 ± 18 | 383 ± 16 | 370 ± 15 | 368 ± 18 | 366 ± 11 | 361 ± 7 | 335 ± 11 | 317 ± 10 | <0.05 |
|  | 20 | 261 ± 8 | 566 ± 95 | 616 ± 45 | 583 ± 19 | 540 ± 14 | 496 ± 15 | 500 ± 19 | 486 ± 74 | 76 ± 11 | 454 ± 11 | 437 ± 6 | <0.05 |
|  | 30 | 253 ± 12 | 551 ± 86 | 696 ± 73 | 677 ± 56 | 682 ± 18 | 633 ± 27 | 610 ± 18 | 588 ± 26 | 581 ± 17 | 552 ± 14 | 520 ± 22 | <0.05 |
|  | 45 | 257 ± 12 | 715 ± 162 | 809 ± 84 | 835 ± 48 | 792 ± 29 | 807 ± 34 | 757 ± 31 | 756 ± 23 | 755 ± 22 | 681 ± 26 | 657 ± 14 | <0.05 |
|  | 60 | 247 ± 7 | 870 ± 133 | 1259 ± 118 | 1195 ± 52 | 1124 ± 42 | 1094 ± 41 | 1045 ± 30 | 1025 ± 24 | 1006 ± 24 | 897 ± 9 | 848 ± 10 | <0.05 |
|  | 75 | 237 ± 8 | 887 ± 129 | 1316 ± 72 | 1276 ± 60 | 1277 ± 35 | 1227 ± 66 | 1169 ± 50 | 1115 ± 45 | 1090 ± 33 | 975 ± 36 | 936 ± 23 | <0.05 |
|  | 90 | 237 ± 7 | 772 ± 109 | 1439 ± 142 | 1578 ± 133 | 1511 ± 83 | 1476 ± 95 | 1394 ± 49 | 1346 ± 36 | 1264 ± 41 | 1174 ± 41 | 1035 ± 28 | <0.05 |
| Met | 0 | 34 ± 1 | 33 ± 2 | 31 ± 2 | 30 ± 2 | 29 ± 2 | 28 ± 2 | 29 ± 2 | 27 ± 2 | 28 ± 2 | 28 ± 1 | 28 ± 1 | NS |
|  | 10 | 33 ± 1 | 32 ± 1 | 29 ± 1 | 29 ± 1 | 28 ± 1 | 28 ± 1 | 28 ± 1 | 27 ± 1 | 27 ± 1 | 26 ± 1 | 26 ± 1 | <0.05 |
|  | 20 | 33 ± 2 | 34 ± 2 | 31 ± 2 | 30 ± 2 | 30 ± 2 | 28 ± 2 | 28 ± 2 | 28 ± 2 | 28 ± 2 | 27 ± 2 | 29 ± 2 | NS |
|  | 30 | 31 ± 1 | 31 ± 1 | 30 ± 1 | 28 ± 1 | 28 ± 1 | 27 ± 1 | 27 ± 1 | 27 ± 1 | 27 ± 1 | 26 ± 1 | 26 ± 1 | <0.05 |
|  | 45 | 31 ± 1 | 31 ± 1 | 30 ± 1 | 28 ± 1 | 26 ± 1 | 27 ± 1 | 25 ± 1 | 25 ± 1 | 26 ± 1 | 24 ± 1 | 25 ± 0 | <0.05 |
|  | 60 | 34 ± 1 | 33 ± 1 | 35 ± 2 | 34 ± 1 | 32 ± 2 | 31 ± 1 | 32 ± 2 | 30 ± 1 | 31 ± 1 | 29 ± 1 | 29 ± 1 | NS |
|  | 75 | 32 ± 1 | 34 ± 1 | 33 ± 1 | 31 ± 1 | 30 ± 1 | 29 ± 1 | 28 ± 2 | 28 ± 1 | 28 ± 1 | 27 ± 1 | 27 ± 1 | <0.05 |
|  | 90 | 30 ± 1 | 32 ± 1 | 30 ± 1 | 30 ± 2 | 29 ± 2 | 29 ± 1 | 28 ± 1 | 26 ± 2 | 25 ± 1 | 25 ± 1 | 24 ± 1 | <0.05 |
| Phe | 0 | 68 ± 3 | 67 ± 3 | 63 ± 2 | 60 ± 3 | 60 ± 3 | 59 ± 2 | 58 ± 2 | 56 ± 2 | 58 ± 3 | 59 ± 1 | 62 ± 1 | NS |
|  | 10 | 68 ± 1 | 68 ± 1 | 62 ± 2 | 63 ± 2 | 61 ± 1 | 60 ± 2 | 60 ± 2 | 60 ± 2 | 60 ± 2 | 58 ± 3 | 57 ± 2 | <0.05 |
|  | 20 | 72 ± 3 | 72 ± 4 | 71 ± 2 | 67 ± 2 | 66 ± 3 | 61 ± 3 | 63 ± 3 | 63 ± 3 | 62 ± 2 | 61 ± 2 | 65 ± 1 | NS |
|  | 30 | 68 ± 3 | 70 ± 2 | 67 ± 3 | 63 ± 3 | 63 ± 3 | 61 ± 3 | 62 ± 3 | 61 ± 4 | 61 ± 2 | 61 ± 2 | 61 ± 3 | NS |
|  | 45 | 65 ± 2 | 66 ± 2 | 62 ± 4 | 59 ± 3 | 57 ± 3 | 58 ± 2 | 55 ± 3 | 56 ± 3 | 57 ± 2 | 56 ± 3 | 57 ± 2 | NS |
|  | 60 | 68 ± 3 | 69 ± 3 | 72 ± 3 | 69 ± 3 | 65 ± 3 | 62 ± 3 | 62 ± 3 | 62 ± 2 | 63 ± 3 | 61 ± 2 | 62 ± 2 | NS |
|  | 75 | 65 ± 3 | 68 ± 3 | 66 ± 2 | 61 ± 3 | 59 ± 2 | 58 ± 2 | 57 ± 2 | 56 ± 2 | 57 ± 2 | 54 ± 3 | 56 ± 1 | <0.05 |
|  | 90 | 57 ± 1 | 61 ± 1 | 59 ± 2 | 58 ± 2 | 55 ± 2 | 55 ± 2 | 53 ± 1 | 51 ± 3 | 49 ± 3 | 50 ± 3 | 47 ± 3 | <0.05 |
| Trp | 0 | 59 ± 5 | 56 ± 5 | 55 ± 5 | 54 ± 5 | 52 ± 5 | 51 ± 5 | 52 ± 4 | 49 ± 4 | 51 ± 3 | 48 ± 4 | 50 ± 3 | NS |
|  | 10 | 64 ± 2 | 64 ± 2 | 60 ± 3 | 62 ± 2 | 62 ± 2 | 59 ± 2 | 60 ± 2 | 59 ± 3 | 60 ± 3 | 54 ± 3 | 51 ± 2 | NS |
|  | 20 | 65 ± 3 | 65 ± 3 | 62 ± 3 | 61 ± 3 | 60 ± 3 | 58 ± 2 | 58 ± 3 | 57 ± 3 | 56 ± 3 | 54 ± 3 | 56 ± 3 | NS |
|  | 30 | 65 ± 7 | 67 ± 6 | 62 ± 7 | 62 ± 7 | 60 ± 6 | 58 ± 5 | 58 ± 6 | 60 ± 6 | 57 ± 6 | 57 ± 5 | 54 ± 5 | NS |
|  | 45 | 64 ± 4 | 62 ± 5 | 58 ± 5 | 58 ± 5 | 57 ± 5 | 56 ± 4 | 53 ± 5 | 52 ± 4 | 54 ± 2 | 52 ± 4 | 53 ± 3 | NS |
|  | 60 | 70 ± 6 | 65 ± 4 | 66 ± 5 | 65 ± 4 | 63 ± 5 | 63 ± 4 | 65 ± 6 | 61 ± 3 | 65 ± 6 | 57 ± 3 | 54 ± 3 | NS |
|  | 75 | 61 ± 4 | 64 ± 5 | 63 ± 4 | 59 ± 5 | 59 ± 3 | 55 ± 4 | 58 ± 4 | 55 ± 3 | 54 ± 3 | 54 ± 2 | 51 ± 2 | NS |
|  | 90 | 58 ± 2 | 59 ± 2 | 56 ± 2 | 56 ± 2 | 54 ± 2 | 55 ± 4 | 53 ± 2 | 53 ± 4 | 50 ± 4 | 50 ± 3 | 45 ± 4 | NS |
| Tyr | 0 | 76 ± 4 | 72 ± 5 | 69 ± 5 | 67 ± 5 | 66 ± 5 | 64 ± 5 | 64 ± 4 | 61 ± 5 | 63 ± 4 | 63 ± 4 | 63 ± 3 | NS |
|  | 10 | 74 ± 4 | 73 ± 4 | 67 ± 4 | 68 ± 5 | 66 ± 4 | 64 ± 4 | 65 ± 4 | 63 ± 5 | 64 ± 4 | 60 ± 5 | 57 ± 5 | NS |
|  | 20 | 77 ± 5 | 76 ± 5 | 76 ± 5 | 72 ± 5 | 69 ± 5 | 65 ± 5 | 67 ± 5 | 66 ± 5 | 66 ± 5 | 64 ± 5 | 66 ± 4 | NS |
|  | 30 | 76 ± 3 | 75 ± 3 | 73 ± 4 | 69 ± 5 | 68 ± 4 | 67 ± 4 | 67 ± 4 | 66 ± 6 | 66 ± 4 | 65 ± 3 | 63 ± 4 | NS |
|  | 45 | 71 ± 3 | 72 ± 3 | 67 ± 4 | 64 ± 3 | 62 ± 3 | 63 ± 2 | 59 ± 3 | 60 ± 3 | 60 ± 3 | 59 ± 3 | 59 ± 2 | <0.05 |
|  | 60 | 75 ± 4 | 77 ± 4 | 81 ± 5 | 78 ± 5 | 74 ± 6 | 72 ± 6 | 71 ± 5 | 71 ± 5 | 72 ± 6 | 69 ± 5 | 68 ± 5 | NS |
|  | 75 | 72 ± 3 | 74 ± 3 | 73 ± 3 | 68 ± 4 | 66 ± 3 | 64 ± 3 | 63 ± 4 | 62 ± 3 | 63 ± 4 | 59 ± 4 | 58 ± 3 | <0.05 |
|  | 90 | 66 ± 1 | 69 ± 1 | 67 ± 4 | 67 ± 3 | 64 ± 3 | 63 ± 3 | 62 ± 2 | 59 ± 4 | 57 ± 4 | 58 ± 3 | 53 ± 4 | <0.05 |

Vales are means ± SE. *P* values were calculated for changes over time within each dose of leucine ingestion by one-way rmANOVA. NS stands for not significant.

**Table S6** Concentrations of plasma amino acids other than BCAAs, methionine, and AAAs in the valine ingestion experiments

| Plasma amino acid | Val ingested (mg/kgBW) | Time after ingestion (min) | | | | | | | | | | | P value |
| --- | --- | --- | --- | --- | --- | --- | --- | --- | --- | --- | --- | --- | --- |
|  |  | 0 | 15 | 30 | 45 | 60 | 75 | 90 | 105 | 120 | 180 | 240 |  |
| (μM) | | | | | | | | | | | | | |
| Ala | 0 | 375 ± 24 | 392 ± 24 | 389 ± 27 | 378 ± 24 | 374 ± 27 | 361 ± 31 | 368 ± 21 | 343 ± 33 | 346 ± 24 | 352 ± 23 | 336 ± 32 | NS |
|  | 10 | 423 ± 18 | 427 ± 15 | 398 ± 18 | 402 ± 28 | 400 ± 26 | 386 ± 26 | 387 ± 32 | 384 ± 32 | 392 ± 26 | 361 ± 31 | 331 ± 35 | NS |
|  | 20 | 417 ± 18 | 417 ± 17 | 418 ± 13 | 399 ± 18 | 392 ± 21 | 361 ± 18 | 375 ± 17 | 369 ± 22 | 362 ± 17 | 354 ± 21 | 358 ± 17 | NS |
|  | 30 | 387 ± 12 | 403 ± 6 | 398 ± 6 | 381 ± 12 | 373 ± 15 | 362 ± 10 | 361 ± 14 | 365 ± 17 | 361 ± 9 | 347 ± 13 | 349 ± 15 | NS |
|  | 45 | 392 ± 18 | 409 ± 18 | 396 ± 23 | 385 ± 23 | 375 ± 20 | 386 ± 13 | 362 ± 18 | 366 ± 20 | 366 ± 17 | 341 ± 24 | 340 ± 22 | NS |
|  | 60 | 422 ± 24 | 437 ± 27 | 474 ± 29 | 466 ± 28 | 448 ± 34 | 439 ± 34 | 433 ± 33 | 430 ± 30 | 439 ± 34 | 402 ± 32 | 409 ± 28 | NS |
|  | 75 | 382 ± 20 | 404 ± 22 | 412 ± 19 | 417 ± 20 | 388 ± 20 | 382 ± 17 | 376 ± 20 | 379 ± 18 | 379 ± 21 | 352 ± 22 | 354 ± 19 | NS |
|  | 90 | 363 ± 28 | 386 ± 27 | 386 ± 31 | 409 ± 34 | 401 ± 34 | 399 ± 23 | 393 ± 25 | 371 ± 28 | 354 ± 20 | 353 ± 15 | 319 ± 34 | NS |
| Arg | 0 | 117 ± 7 | 115 ± 9 | 109 ± 7 | 108 ± 8 | 106 ± 7 | 103 ± 7 | 104 ± 6 | 99 ± 8 | 101 ± 8 | 101 ± 7 | 103 ± 5 | NS |
|  | 10 | 109 ± 4 | 111 ± 5 | 101 ± 3 | 103 ± 6 | 100 ± 5 | 98 ± 5 | 95 ± 6 | 99 ± 5 | 98 ± 6 | 95 ± 6 | 91 ± 5 | NS |
|  | 20 | 101 ± 6 | 105 ± 7 | 104 ± 5 | 101 ± 7 | 100 ± 8 | 93 ± 5 | 96 ± 7 | 97 ± 6 | 96 ± 6 | 91 ± 6 | 97 ± 7 | NS |
|  | 30 | 96 ± 4 | 98 ± 5 | 97 ± 4 | 96 ± 4 | 93 ± 4 | 90 ± 4 | 92 ± 4 | 92 ± 5 | 92 ± 3 | 97 ± 1 | 290 ± 5 | NS |
|  | 45 | 99 ± 5 | 102 ± 5 | 96 ± 7 | 93 ± 7 | 90 ± 6 | 93 ± 5 | 89 ± 5 | 91 ± 7 | 92 ± 5 | 88 ± 4 | 90 ± 5 | NS |
|  | 60 | 97 ± 5 | 103 ± 6 | 109 ± 6 | 107 ± 5 | 103 ± 6 | 104 ± 5 | 101 ± 5 | 102 ± 5 | 103 ± 5 | 100 ± 4 | 101 ± 6 | NS |
|  | 75 | 101 ± 6 | 106 ± 5 | 105 ± 4 | 104 ± 4 | 99 ± 4 | 98 ± 5 | 97 ± 5 | 97 ± 5 | 97 ± 5 | 95 ± 7 | 96 ± 5 | NS |
|  | 90 | 94 ± 3 | 100 ± 4 | 98 ± 4 | 101 ± 6 | 100 ± 5 | 101 ± 4 | 99 ± 2 | 96 ± 6 | 94 ± 5 | 91 ± 4 | 88 ± 5 | NS |
| Asn | 0 | 54 ± 4 | 52 ± 4 | 53 ± 4 | 51 ± 4 | 51 ± 4 | 49 ± 4 | 50 ± 3 | 47 ± 4 | 47 ± 3 | 49 ± 3 | 49 ± 3 | NS |
|  | 10 | 50 ± 2 | 49 ± 2 | 47 ± 3 | 49 ± 2 | 47 ± 2 | 46 ± 2 | 47 ± 2 | 47 ± 2 | 48 ± 3 | 46 ± 4 | 44 ± 3 | NS |
|  | 20 | 51 ± 3 | 54 ± 3 | 54 ± 2 | 52 ± 3 | 51 ± 3 | 51 ± 2 | 51 ± 3 | 52 ± 3 | 50 ± 3 | 49 ± 3 | 54 ± 3 | NS |
|  | 30 | 48 ± 3 | 50 ± 2 | 51 ± 3 | 48 ± 4 | 49 ± 3 | 47 ± 3 | 48 ± 3 | 48 ± 3 | 50 ± 3 | 48 ± 3 | 49 ± 3 | NS |
|  | 45 | 47 ± 4 | 47 ± 3 | 45 ± 3 | 45 ± 3 | 44 ± 3 | 47 ± 1 | 45 ± 4 | 45 ± 3 | 46 ± 3 | 44 ± 3 | 45 ± 4 | NS |
|  | 60 | 56 ± 3 | 59 ± 2 | 62 ± 3 | 62 ± 4 | 61 ± 5 | 59 ± 5 | 61 ± 2 | 62 ± 4 | 62 ± 4 | 59 ± 5 | 60 ± 4 | NS |
|  | 75 | 56 ± 4 | 60 ± 3 | 61 ± 4 | 59 ± 5 | 55 ± 4 | 59 ± 2 | 59 ± 4 | 60 ± 4 | 61 ± 4 | 58 ± 5 | 59 ± 3 | NS |
|  | 90 | 49 ± 2 | 53 ± 2 | 54 ± 3 | 57 ± 3 | 56 ± 4 | 56 ± 4 | 56 ± 3 | 54 ± 4 | 52 ± 4 | 53 ± 3 | 50 ± 4 | NS |
| Asp | 0 | 0.3 ± 0.3 | 0.5 ± 0.3 | 0.3 ± 0.4 | 0.4 ± 0.3 | 0.3 ± 0.4 | 0.3 ± 0.3 | 0.3 ± 0.3 | 0.4 ± 0.3 | 0.9 ± 0.6 | 0.4 ± 0.3 | 0.4 ± 0.3 | NS |
|  | 10 | 1.2 ± 0.2 | 1.3 ± 0.2 | 1.1 ± 0.2 | 1.1 ± 0.1 | 1.3 ± 0.1 | 1.1 ± 0.1 | 1.1 ± 0.1 | 1.2 ± 0.1 | 1.3 ± 0.2 | 1.2 ± 0.1 | 1.1 ± 0.1 | NS |
|  | 20 | 2.0 ± 0.3 | 2.2 ± 0.2 | 2.5 ± 0.2 | 2.1 ± 0.1 | 1.9 ± 0.2 | 1.9 ± 0.2 | 2.3 ± 0.2 | 1.9 ± 0.2 | 1.7 ± 0.2 | 2.2 ± 0.2 | 1.4 ± 0.2 | NS |
|  | 30 | 1.4 ± 0.2 | 1.8 ± 0.2 | 1.5 ± 0.2 | 1.7 ± 0.3 | 1.2 ± 0.0 | 1.6 ± 0.2 | 1.4 ± 0.1 | 1.4 ± 0.2 | 1.3 ± 0.2 | 1.5 ± 0.2 | 1.5 ± 0.2 | NS |
|  | 45 | 1.7 ± 0.5 | 2.0 ± 0.5 | 1.3 ± 0.2 | 1.0 ± 0.1 | 1.0 ± 0.2 | 1.7 ± 0.5 | 1.1 ± 0.3 | 1.2 ± 0.1 | 1.5 ± 0.3 | 1.7 ± 0.3 | 1.3 ± 0.3 | NS |
|  | 60 | 2.0 ± 0.4 | 2.7 ± 0.5 | 2.2 ± 0.3 | 2.6 ± 0.4 | 1.4 ± 0.3 | 2.1 ± 0.3 | 1.9 ± 0.4 | 1.1 ± 0.3 | 2.0 ± 0.4 | 1.7 ± 0.1 | 1.8v0.2 | NS |
|  | 75 | 1.2 ± 0.2 | 1.1 ± 0.1 | 1.3 ± 0.0 | 1.7 ± 0.4 | 1.2 ± 0.2 | 1.5 ± 0.3 | 2.7 ± 0.2 | 1.0 ± 1.3 | 1.4 ± 0.4 | 1.5 ± 0.3 | 1.3 ± 0.2 | NS |
|  | 90 | 1.6 ± 0.2 | 1.6 ± 0.1 | 1.2 ± 0.1 | 1.2 ± 0.1 | 1.4 ± 0.2 | 1.4 ± 0.2 | 1.7 ± 0.3 | 2.0 ± 0.2 | 1.6 ± 0.3 | 1.8 ± 0.3 | 1.6 ± 0.2 | NS |
| Cys | 0 | 51 ± 1 | 49 ± 2 | 50 ± 2 | 50 ± 2 | 49 ± 2 | 49 ± 2 | 49 ± 2 | 49 ± 2 | 50 ± 3 | 48 ± 2 | 48 ± 2 | NS |
|  | 10 | 54 ± 2 | 54 ± 1 | 51 ± 1 | 54 ± 2 | 54 ± 2 | 54 ± 2 | 54 ± 2 | 55 ± 1 | 53 ± 1 | 52 ± 2 | 50 ± 2 | NS |
|  | 20 | 55 ± 2 | 56 ± 2 | 56 ± 2 | 55 ± 2 | 55 ± 2 | 54 ± 3 | 55 ± 2 | 54 ± 2 | 54 ± 2 | 56 ± 2 | 53 ± 2 | NS |
|  | 30 | 56 ± 2 | 57 ± 2 | 57 ± 3 | 55 ± 3 | 56 ± 3 | 57 ± 3 | 56 ± 2 | 55 ± 3 | 56 ± 2 | 57 ± 2 | 56 ± 3 | NS |
|  | 45 | 54 ± 2 | 55 ± 2 | 52 ± 3 | 52 ± 3 | 52 ± 2 | 54 ± 1 | 51 ± 2 | 52 ± 2 | 53 ± 1 | 53 ± 1 | 54 ± 1 | NS |
|  | 60 | 52 ± 1 | 56 ± 1 | 58 ± 2 | 57 ± 2 | 57 ± 2 | 57 ± 1 | 56 ± 2 | 56 ± 2 | 57 ± 2 | 57 ± 2 | 56 ± 2 | NS |
|  | 75 | 54 ± 2 | 56 ± 2 | 57 ± 2 | 55 ± 2 | 55 ± 2 | 56 ± 2 | 55 ± 1 | 55 ± 1 | 54 ± 2 | 54 ± 2 | 54 ± 2 | NS |
|  | 90 | 53 ± 1 | 56 ± 2 | 56 ± 3 | 57 ± 2 | 55 ± 2 | 56 ± 3 | 56 ± 1 | 55 ± 2 | 53 ± 2 | 56 ± 2 | 53 ± 3 | NS |
| Gln | 0 | 624 ± 18 | 621 ± 28 | 614 ± 27 | 600 ± 27 | 602 ± 31 | 588 ± 31 | 592 ± 27 | 552 ± 40 | 582 ± 33 | 620 ± 25 | 630 ± 14 | NS |
|  | 10 | 614 ± 14 | 597 ± 11 | 570 ± 75 | 94 ± 13 | 587 ± 95 | 77 ± 18 | 581 ± 22 | 582 ± 15 | 599 ± 17 | 591 ± 23 | 588 ± 22 | NS |
|  | 20 | 585 ± 16 | 580 ± 17 | 589 ± 15 | 576 ± 18 | 581 ± 17 | 541 ± 14 | 564 ± 12 | 571 ± 17 | 569 ± 13 | 586 ± 14 | 620 ± 14 | NS |
|  | 30 | 564 ± 16 | 571 ± 13 | 579 ± 12 | 561 ± 8 | 550 ± 17 | 546 ± 12 | 557 ± 11 | 563 ± 14 | 573 ± 6 | 571 ± 5 | 595 ± 9 | NS |
|  | 45 | 570 ± 21 | 583 ± 19 | 579 ± 39 | 565 ± 35 | 551 ± 30 | 566 ± 24 | 546 ± 29 | 561 ± 23 | 569 ± 16 | 564 ± 19 | 588 ± 17 | NS |
|  | 60 | 567 ± 15 | 545 ± 12 | 598 ± 16 | 605 ± 85 | 79 ± 12 | 572 ± 12 | 569 ± 6 | 575 ± 9 | 595 ± 4 | 581 ± 7 | 623 ± 13 | <0.05 |
|  | 75 | 572 ± 13 | 594 ± 12 | 596 ± 8 | 595 ± 15 | 572 ± 13 | 570 ± 10 | 569 ± 12 | 572 ± 10 | 587 ± 13 | 585 ± 20 | 616 ± 12 | NS |
|  | 90 | 555 ± 18 | 574 ± 17 | 577 ± 12 | 607 ± 28 | 602 ± 29 | 588 ± 13 | 580 ± 21 | 558 ± 28 | 553 ± 23 | 574 ± 22 | 565 ± 21 | NS |
| Glu | 0 | 26 ± 3 | 27 ± 6 | 33 ± 5 | 35 ± 4 | 40 ± 7 | 38 ± 7 | 38 ± 5 | 41 ± 6 | 42 ± 6 | 34 ± 4 | 34 ± 4 | NS |
|  | 10 | 36 ± 4 | 35 ± 4 | 36 ± 5 | 38 ± 4 | 37 ± 2 | 36 ± 3 | 35 ± 3 | 39 ± 3 | 34 ± 2 | 34 ± 2 | 34 ± 3 | NS |
|  | 20 | 46 ± 4 | 48 ± 3 | 46 ± 3 | 45 ± 3 | 43 ± 4 | 46 ± 2 | 46 ± 4 | 42 ± 4 | 42 ± 3 | 46 ± 3 | 34 ± 4 | NS |
|  | 30 | 40 ± 5 | 37 ± 5 | 42 ± 6 | 40 ± 1 | 42 ± 2 | 45 ± 4 | 39 ± 2 | 35 ± 3 | 36 ± 3 | 43 ± 3 | 37 ± 5 | NS |
|  | 45 | 41±5 | 39 ± 6 | 35 ± 4 | 37 ± 4 | 36 ± 4 | 40 ± 5 | 38 ± 4 | 38 ± 4 | 42 ± 5 | 41 ± 4 | 38 ± 5 | NS |
|  | 60 | 41 ± 2 | 48 ± 3 | 43 ± 1 | 37 ± 1 | 40 ± 2 | 44 ± 3 | 38 ± 3 | 38 ± 3 | 38 ± 2 | 43 ± 4 | 35 ± 3 | NS |
|  | 75 | 40 ± 1 | 38 ± 2 | 40 ± 2 | 44 ± 2 | 38 ± 2 | 40 ± 4 | 35 ± 4 | 37 ± 4 | 33 ± 3 | 40 ± 2 | 41 ± 5 | NS |
|  | 90 | 39 ± 3 | 40 ± 2 | 42 ± 1 | 37 ± 1 | 36 ± 2 | 38 ± 5 | 42 ± 6 | 45 ± 6 | 48 ± 6 | 48 ± 7 | 42 ± 4 | NS |
| (Table S6-2) | | | | | | | | | | | | | |
| (μM) | | | | | | | | | | | | | |
| Gyl | 0 | 266 ± 7 | 266 ± 9 | 264 ± 5 | 259 ± 7 | 258 ± 8 | 254 ± 7 | 257 ± 9 | 242 ± 9 | 252 ± 1 | 2255 ± 7 | 255 ± 4 | NS |
|  | 10 | 257 ± 9 | 254 ± 9 | 241 ± 7 | 251 ± 7 | 251 ± 8 | 247 ± 8 | 248 ± 16 | 249 ± 8 | 252 ± 7 | 236 ± 6 | 227 ± 8 | NS |
|  | 20 | 257 ± 9 | 255 ± 8 | 262 ± 9 | 256 ± 9 | 260 ± 11 | 245 ± 6 | 252 ± 9 | 253 ± 7 | 253 ± 5 | 251 ± 8 | 257 ± 9 | NS |
|  | 30 | 234 ± 10 | 239 ± 11 | 243 ± 9 | 235 ± 8 | 235 ± 6 | 236 ± 8 | 238 ± 9 | 236 ± 9 | 241 ± 9 | 236 ± 8 | 237 ± 8 | NS |
|  | 45 | 248 ± 10 | 254 ± 11 | 247 ± 14 | 245 ± 14 | 241 ± 12 | 248 ± 11 | 236 ± 7 | 243 ± 13 | 246 ± 9 | 241 ± 6 | 245 ± 9 | NS |
|  | 60 | 259 ± 11 | 260 ± 12 | 281 ± 15 | 283 ± 13 | 273 ± 10 | 275 ± 11 | 272 ± 10 | 274 ± 13 | 279 ± 10 | 268 ± 10 | 277 ± 11 | NS |
|  | 75 | 245 ± 8 | 254 ± 9 | 255 ± 7 | 259 ± 8 | 251 ± 2 | 246 ± 5 | 245±7 | 247 ± 8 | 250 ± 8 | 240 ± 5 | 246 ± 6 | NS |
|  | 90 | 230 ± 14 | 241 ± 15 | 238 ± 11 | 250 ± 19 | 250 ± 19 | 249 ± 4 | 248 ± 14 | 235 ± 10 | 232 ± 7 | 237 ± 6 | 226 ± 11 | NS |
| His | 0 | 90 ± 3 | 88 ± 4 | 88 ± 2 | 85 ± 3 | 86 ± 3 | 85 ± 3 | 87 ± 3 | 82 ± 4 | 85 ± 4 | 88 ± 3 | 88 ± 1 | NS |
|  | 10 | 89 ± 2 | 88 ± 2 | 84 ± 2 | 86 ± 1 | 87 ± 1 | 85 ± 2 | 85 ± 4 | 85 ± 2 | 89 ± 2 | 85 ± 1 | 83 ± 2 | NS |
|  | 20 | 86 ± 3 | 85 ± 2 | 88 ± 3 | 85 ± 2 | 85 ± 3 | 82 ± 3 | 84 ± 4 | 84 ± 2 | 84 ± 2 | 85 ± 3 | 88 ± 4 | NS |
|  | 30 | 84 ± 2 | 84 ± 2 | 85 ± 2 | 84 ± 2 | 83 ± 2 | 82 ± 2 | 82 ± 3 | 83 ± 3 | 85 ± 2 | 84 ± 3 | 85 ± 4 | NS |
|  | 45 | 81 ± 2 | 85 ± 3 | 83 ± 4 | 80 ± 3 | 79 ± 3 | 81 ± 3 | 78 ± 4 | 79 ± 4 | 81 ± 2 | 79 ± 4 | 82 ± 2 | NS |
|  | 60 | 86 ± 4 | 83 ± 2 | 91 ± 3 | 89 ± 2 | 89 ± 3 | 85 ± 2 | 84 ± 3 | 87 ± 3 | 88 ± 2 | 87 ± 2 | 89 ± 2 | NS |
|  | 75 | 80 ± 3 | 83 ± 3 | 85 ± 2 | 85 ± 1 | 82 ± 1 | 81 ± 2 | 82 ± 2 | 81 ± 2 | 82 ± 1 | 80 ± 3 | 83 ± 2 | NS |
|  | 90 | 78 ± 3 | 81 ± 3 | 82 ± 3 | 86 ± 3 | 83 ± 2 | 83 ± 4 | 82 ± 2 | 80 ± 5 | 79 ± 5 | 81 ± 5 | 77 ± 3 | NS |
| Lys | 0 | 220 ± 8 | 219 ± 11 | 216 ± 10 | 208 ± 10 | 208 ± 11 | 204 ± 11 | 207 ± 9 | 196 ± 11 | 202 ± 10 | 204 ± 9 | 206 ± 6 | NS |
|  | 10 | 210 ± 11 | 210 ± 11 | 196 ± 9 | 202 ± 8 | 202 ± 7 | 198 ± 6 | 198 ± 10 | 199 ± 5 | 204 ± 9 | 193 ± 11 | 185 ± 8 | NS |
|  | 20 | 202 ± 6 | 203 ± 5 | 208 ± 4 | 199 ± 6 | 199 ± 7 | 189 ± 6 | 198 ± 7 | 198 ± 7 | 197 ± 7 | 195 ± 8 | 204 ± 9 | NS |
|  | 30 | 190 ± 6 | 195 ± 5 | 192 ± 5 | 185 ± 3 | 184 ± 6 | 183 ± 3 | 185 ± 4 | 184 ± 4 | 187 ± 3 | 184 ± 4 | 183 ± 4 | NS |
|  | 45 | 196 ± 8 | 200 ± 9 | 191 ± 15 | 186 ± 14 | 182 ± 11 | 187 ± 9 | 180 ± 12 | 183 ± 11 | 186 ± 8 | 179 ± 8 | 184 ± 7 | NS |
|  | 60 | 179 ± 4 | 182 ± 5 | 194 ± 4 | 190 ± 7 | 185 ± 7 | 186 ± 6 | 184 ± 6 | 185 ± 8 | 190 ± 6 | 185 ± 7 | 189 ± 6 | NS |
|  | 75 | 175 ± 8 | 183 ± 8 | 181 ± 8 | 180 ± 8 | 170 ± 8 | 172 ± 8 | 171 ± 8 | 171 ± 8 | 173 ± 9 | 167 ± 12 | 173 ± 9 | NS |
|  | 90 | 195 ± 10 | 202 ± 9 | 196 ± 6 | 202 ± 9 | 200 ± 11 | 201 ± 12 | 198 ± 10 | 193 ± 16 | 187 ± 15 | 190 ± 12 | 177 ± 12 | NS |
| Pro | 0 | 147 ± 8 | 147 ± 8 | 144 ± 5 | 145 ± 7 | 141 ± 7 | 142 ± 7 | 145 ± 9 | 137 ± 9 | 137 ± 8 | 134 ± 7 | 130 ± 6 | NS |
|  | 10 | 169 ± 17 | 167 ± 17 | 155 ± 12 | 157 ± 18 | 157 ± 19 | 155 ± 16 | 151 ± 20 | 150 ± 18 | 152 ± 17 | 142 ± 17 | 137 ± 18 | NS |
|  | 20 | 159 ± 8 | 159 ± 9 | 157 ± 10 | 152 ± 9 | 151 ± 11 | 143 ± 9 | 146 ± 10 | 141 ± 10 | 142 ± 9 | 139 ± 11 | 142 ± 11 | NS |
|  | 30 | 154 ± 12 | 153 ± 10 | 150 ± 11 | 146 ± 11 | 144 ± 10 | 146 ± 12 | 141 ± 12 | 140 ± 13 | 143 ± 11 | 140 ± 11 | 138 ± 12 | NS |
|  | 45 | 164 ± 13 | 164 ± 12 | 152 ± 12 | 151 ± 12 | 150 ± 13 | 152 ± 11 | 143 ± 7 | 146 ± 14 | 146 ± 10 | 142 ± 8 | 141 ± 12 | NS |
|  | 60 | 150 ± 7 | 159 ± 11 | 168 ± 15 | 165 ± 11 | 158 ± 12 | 160 ± 14 | 154 ± 13 | 155 ± 15 | 161 ± 15 | 150 ± 11 | 149 ± 12 | NS |
|  | 75 | 155 ± 16 | 159 ± 14 | 154 ± 13 | 153 ± 12 | 146 ± 13 | 144 ± 12 | 142 ± 12 | 144 ± 14 | 142 ± 13 | 130 ± 12 | 133 ± 12 | NS |
|  | 90 | 141 ± 11 | 146 ± 12 | 145 ± 13 | 147 ± 14 | 147 ± 14 | 144 ± 10 | 141 ± 12 | 136 ± 11 | 133 ± 10 | 132±9 | 124 ± 11 | NS |
| Ser | 0 | 129 ± 6 | 129 ± 6 | 129 ± 6 | 126 ± 6 | 125 ± 5 | 124 ± 5 | 126 ± 7 | 121 ± 6 | 125 ± 7 | 125 ± 5 | 126 ± 5 | NS |
|  | 10 | 119 ± 6 | 120 ± 6 | 112 ± 5 | 116 ± 6 | 116 ± 5 | 114 ± 5 | 115 ± 8 | 117 ± 4 | 115 ± 5 | 113 ± 7 | 113 ± 7 | NS |
|  | 20 | 125 ± 6 | 129 ± 6 | 130 ± 7 | 126 ± 6 | 125 ± 6 | 122 ± 6 | 124 ± 7 | 124 ± 6 | 124 ± 5 | 129 ± 6 | 126 ± 6 | NS |
|  | 30 | 116 ± 8 | 120 ± 7 | 121 ± 6 | 115 ± 6 | 120 ± 8 | 119 ± 5 | 118 ± 7 | 116 ± 4 | 118 ± 6 | 124 ± 7 | 122 ± 6 | NS |
|  | 45 | 123 ± 8 | 127 ± 8 | 122 ± 10 | 124 ± 8 | 120 ± 8 | 126 ± 7 | 121 ± 8 | 123 ± 7 | 125 ± 7 | 125 ± 9 | 129 ± 7 | NS |
|  | 60 | 124 ± 10 | 132 ± 10 | 139 ± 10 | 134 ± 11 | 135 ± 11 | 136 ± 9 | 136 ± 10 | 137 ± 9 | 139 ± 8 | 140 ± 8 | 138 ± 9 | NS |
|  | 75 | 121 ± 7 | 131 ± 8 | 135 ± 7 | 127 ± 9 | 129 ± 8 | 131 ± 7 | 129 ± 7 | 131±7 | 132 ± 7 | 131 ± 4 | 135 ± 6 | NS |
|  | 90 | 113 ± 7 | 120±8 | 122 ± 5 | 119 ± 9 | 126 ± 9 | 126 ± 2 | 127 ± 6 | 127 ± 7 | 122 ± 6 | 127 ± 6 | 123 ± 7 | NS |
| Thr | 0 | 160 ± 8 | 159 ± 10 | 157 ± 9 | 151 ± 9 | 150 ± 9 | 147 ± 10 | 149 ± 9 | 142 ± 10 | 146 ± 10 | 149 ± 9 | 149 ± 7 | NS |
|  | 10 | 148 ± 8 | 147 ± 8 | 138 ± 9 | 142 ± 10 | 142 ± 9 | 138 ± 9 | 139 ± 10 | 140 ± 10 | 142 ± 10 | 135 ± 12 | 128 ± 11 | NS |
|  | 20 | 147 ± 11 | 149 ± 11 | 152 ± 10 | 147 ± 11 | 145 ± 12 | 138 ± 11 | 143 ± 10 | 144 ± 12 | 143 ± 11 | 143 ± 12 | 147 ± 11 | NS |
|  | 30 | 132 ± 71 | 36 ± 6 | 136 ± 7 | 132 ± 9 | 131 ± 7 | 130 ± 7 | 131 ± 8 | 131 ± 9 | 132 ± 7 | 131 ± 7 | 132 ± 7 | NS |
|  | 45 | 151 ± 6 | 156 ± 6 | 150 ± 8 | 148 ± 8 | 146 ± 7 | 150 ± 5 | 143 ± 5 | 148 ± 8 | 149 ± 7 | 144 ± 4 | 148 ± 7 | NS |
|  | 60 | 148 ± 5 | 157 ± 5 | 171 ± 6 | 171 ± 8 | 165 ± 9 | 164 ± 9 | 163 ± 8 | 165 ± 10 | 168 ± 8 | 162 ± 9 | 164 ± 8 | NS |
|  | 75 | 141 ± 11 | 152 ± 9 | 157 ± 12 | 157 ± 14 | 150 ± 14 | 150 ± 12 | 148 ± 14 | 151 ± 11 | 152 ± 13 | 144 ± 14 | 148 ± 10 | NS |
|  | 90 | 132 ± 6 | 142 ± 5 | 144 ± 9 | 153 ± 10 | 151 ± 11 | 152 ± 9 | 151 ± 8 | 146 ± 9 | 142 ± 8 | 144 ± 5 | 134 ± 11 | NS |

Vales are means ± SE. *P* values were calculated for changes over time within each dose of leucine ingestion by one-way rmANOVA. NS stands for not significant.

**Table S7** Concentrations of plasma BCAAs, methionine, and AAAs in the mixed BCAA ingestion experiments

| Plasma amino acid | Mixed BCAA ingested (mg/kg BW) | Time after ingestion (min) | | | | | | | | | | | P value |
| --- | --- | --- | --- | --- | --- | --- | --- | --- | --- | --- | --- | --- | --- |
|  |  | 0 | 15 | 30 | 45 | 60 | 75 | 90 | 105 | 120 | 180 | 240 |  |
| (μM) | | | | | | | | | | | | | |
| Leu | 0 | 125 ± 7 | 117 ± 6 | 112 ± 6 | 106 ± 7 | 105 ± 6 | 104 ± 6 | 106 ± 6 | 112 ± 6 | 106 ± 12 | 113 ± 3 | 118 ± 2 | NS |
|  | 63 | 150 ± 5 | 266 ± 25 | 388 ± 30 | 379 ± 22 | 339 ± 15 | 305 ± 8 | 280 ± 6 | 260 ± 10 | 245 ± 12 | 195 ± 11 | 173 ± 10 | <0.05 |
|  | 94.5 | 145 ± 5 | 355 ± 52 | 527 ± 57 | 538 ± 49 | 499 ± 36 | 403 ± 22 | 341 ± 9 | 303 ± 11 | 281 ± 8 | 203 ± 11 | 180 ± 5 | <0.05 |
| Ile | 0 | 63 ± 4 | 58 ± 4 | 55 ± 4 | 52 ± 4 | 51 ± 3 | 50 ± 3 | 51 ± 3 | 48 ± 3 | 50 ± 3 | 52 ± 2 | 54 ± 2 | NS |
|  | 63 | 76 ± 4 | 134 ± 13 | 198 ± 17 | 190 ± 12 | 166 ± 9 | 149 ± 6 | 134 ± 5 | 122 ± 8 | 113 ± 8 | 86 ± 8 | 76 ± 6 | <0.05 |
|  | 94.5 | 74 ± 4 | 173 ± 24 | 264 ± 27 | 272 ± 25 | 248 ± 19 | 191 ± 12 | 156 ± 6 | 135 ± 6 | 122 ± 5 | 83 ± 7 | 72 ± 3 | <0.05 |
| Val | 0 | 248 ± 9 | 239 ± 9 | 236 ± 8 | 230 ± 8 | 226 ± 7 | 223 ± 8 | 225 ± 8 | 215 ± 8 | 223 ± 7 | 225 ± 7 | 230 ± 7 | NS |
|  | 63 | 281 ± 9 | 360 ± 16 | 483 ± 29 | 497 ± 27 | 474 ± 19 | 444 ± 13 | 426 ± 11 | 405 ± 16 | 393 ± 18 | 346 ± 16 | 323 ± 17 | <0.05 |
|  | 94.5 | 277 ± 10 | 399 ± 33 | 558 ± 48 | 610 ± 51 | 626 ± 44 | 555 ± 24 | 491 ± 12 | 456 ± 16 | 440 ± 9 | 365 ± 13 | 340 ± 9 | <0.05 |
| Met | 0 | 34 ± 1 | 33 ± 2 | 31 ± 2 | 30 ± 2 | 29 ± 2 | 28 ± 2 | 29 ± 2 | 27 ± 2 | 28 ± 2 | 28 ± 1 | 28 ± 1 | NS |
|  | 63 | 36 ± 1 | 37 ± 1 | 36 ± 1 | 35 ± 1 | 32 ± 1 | 35 ± 1 | 34 ± 4 | 31 ± 4 | 31 ± 4 | 29 ± 5 | 28 ± 4 | NS |
|  | 94.5 | 36 ± 1 | 36 ± 1 | 35 ± 1 | 33 ± 2 | 31 ± 2 | 27 ± 2 | 26 ± 2 | 24 ± 1 | 23 ± 2 | 21 ± 1 | 22 ± 1 | <0.05 |
| Phe | 0 | 68 ± 3 | 67 ± 3 | 63 ± 2 | 60 ± 3 | 60 ± 3 | 59 ± 2 | 58 ± 2 | 56 ± 2 | 58 ± 3 | 59 ± 1 | 62 ± 1 | NS |
|  | 63 | 76 ± 3 | 79 ± 2 | 76 ± 2 | 70 ± 2 | 65 ± 2 | 63 ± 2 | 62 ± 2 | 58 ± 3 | 57 ± 2 | 56 ± 2 | 57 ± 2 | <0.05 |
|  | 94.5 | 76 ± 3 | 77 ± 3 | 72 ± 3 | 67 ± 3 | 63 ± 3 | 56 ± 3 | 53 ± 2 | 51 ± 3 | 50 ± 3 | 48 ± 2 | 51 ± 2 | <0.05 |
| Trp | 0 | 59 ± 5 | 56 ± 5 | 55 ± 5 | 54 ± 5 | 52 ± 5 | 51 ± 5 | 52 ± 4 | 49 ± 4 | 51 ± 3 | 48 ± 4 | 50 ± 3 | NS |
|  | 63 | 65 ± 4 | 66 ± 4 | 62 ± 3 | 57 ± 3 | 57 ± 3 | 66 ± 3 | 52 ± 11 | 63 ± 3 | 59 ± 12 | 61 ± 9 | 57 ± 12 | NS |
|  | 94.5 | 73 ± 4 | 72 ± 4 | 66 ± 4 | 63 ± 3 | 61 ± 3 | 59 ± 4 | 54 ± 3 | 53 ± 3 | 53 ± 3 | 49 ± 3 | 51 ± 2 | <0.05 |
| Tyr | 0 | 76 ± 4 | 72 ± 5 | 69 ± 5 | 67 ± 5 | 66 ± 5 | 64 ± 5 | 64 ± 4 | 61 ± 5 | 63 ± 4 | 63 ± 4 | 63 ± 3 | NS |
|  | 63 | 80 ± 4 | 83 ± 4 | 81 ± 4 | 76 ± 4 | 70 ± 4 | 68 ± 3 | 66 ± 3 | 61 ± 5 | 60 ± 4 | 56 ± 3 | 54 ± 3 | <0.05 |
|  | 94.5 | 81 ± 4 | 81 ± 4 | 78 ± 4 | 74 ± 4 | 71 ± 5 | 63 ± 5 | 60 ± 4 | 57 ± 4 | 55 ± 4 | 49 ± 4 | 48 ± 3 | <0.05 |

Values are means ± SE. *P* values were calculated for changes over time within each dose of leucine ingestion by one-way rmANOVA. NS stands for not significant.

**Table S8** Concentrations of plasma amino acids other than BCAAs, methionine, and AAAs in the mixed BCAA ingestion experimensts

| Plasma amino acid | Mixed BCAA ingested (mg/kg BW) | Time after ingestion (min) | | | | | | | | | | | P value |
| --- | --- | --- | --- | --- | --- | --- | --- | --- | --- | --- | --- | --- | --- |
|  |  | 0 | 15 | 30 | 45 | 60 | 75 | 90 | 105 | 120 | 180 | 240 |  |
| (μM) | | | | | | | | | | | | | |
| Ala | 0 | 375 ± 24 | 392 ± 24 | 389 ± 27 | 378 ± 24 | 374 ± 27 | 361 ± 31 | 368 ± 21 | 343 ± 33 | 346 ± 24 | 352 ± 23 | 336 ± 32 | NS |
|  | 63 | 444 ± 22 | 460 ± 21 | 448 ± 23 | 429 ± 19 | 419 ± 22 | 413 ± 17 | 414 ± 15 | 396 ± 30 | 395 ± 24 | 401 ± 18 | 372 ± 24 | NS |
|  | 94.5 | 433 ± 16 | 432 ± 21 | 408 ± 24 | 401 ± 22 | 399 ± 18 | 377 ± 18 | 375 ± 18 | 368 ± 20 | 374 ± 19 | 361 ± 19 | 356 ± 20 | NS |
| Arg | 0 | 117 ± 7 | 115 ± 9 | 109 ± 7 | 108 ± 8 | 106 ± 7 | 103 ± 7 | 104 ± 6 | 99 ± 8 | 101 ± 8 | 101 ± 7 | 103 ± 5 | NS |
|  | 63 | 116 ± 5 | 127 ± 5 | 129 ± 6 | 127 ± 5 | 124 ± 6 | 121 ± 4 | 122 ± 4 | 114 ± 6 | 112 ± 5 | 107 ± 3 | 100 ± 3 | <0.05 |
|  | 94.5 | 115 ± 6 | 120 ± 7 | 125 ± 8 | 128 ± 9 | 128 ± 8 | 119 ± 8 | 115 ± 7 | 110 ± 9 | 110 ± 9 | 97 ± 7 | 93 ± 5 | NS |
| Asn | 0 | 54 ± 4 | 52 ± 4 | 53 ± 4 | 51 ± 4 | 51 ± 4 | 49 ± 4 | 50 ± 3 | 47 ± 4 | 47 ± 3 | 49 ± 3 | 49 ± 3 | NS |
|  | 63 | 51 ± 1 | 55 ± 1 | 53 ± 2 | 51 ± 1 | 49 ± 2 | 48 ± 2 | 48 ± 1 | 46 ± 2 | 45 ± 2 | 46 ± 2 | 45 ± 2 | <0.05 |
|  | 94.5 | 52 ± 2 | 51 ± 2 | 49 ± 2 | 47 ± 2 | 46 ± 3 | 43 ± 3 | 44 ± 2 | 42 ± 3 | 43 ± 2 | 41 ± 3 | 42 ± 2 | <0.05 |
| Asp | 0 | 0.3 ± 0.3 | 0.5 ± 0.3 | 0.3 ± 0.4 | 0.4 ± 0.3 | 0.3 ± 0.4 | 0.3 ± 0.3 | 0.3 ± 0.3 | 0.4 ± 0.3 | 0.9 ± 0.6 | 0.4 ± 0.3 | 0.4 ± 0.3 | NS |
|  | 63 | 1.1 ± 0.2 | 1.1 ± 0.1 | 1.2 ± 0.1 | 1.1 ± 0.1 | 1.2 ± 0.1 | 1.1 ± 0.1 | 1.1 ± 0.0 | 1.6 ± 0.1 | 1.4 ± 0.2 | 1.2 ± 0.1 | 1.1 ± 0.1 | NS |
|  | 94.5 | 1.7 ± 0.2 | 1.3 ± 0.3 | 1.4 ± 0.2 | 1.3 ± 0.2 | 1.4 ± 0.2 | 1.4 ± 0.1 | 2.3 ± 0.3 | 2.1 ± 0.2 | 1.3 ± 0.6 | 1.3 ± 0.2 | 1.4 ± 0.2 | NS |
| Cys | 0 | 51 ± 1 | 49 ± 2 | 50 ± 2 | 50 ± 2 | 49 ± 2 | 49 ± 2 | 49 ± 2 | 49 ± 2 | 50 ± 3 | 48 ± 2 | 48 ± 2 | NS |
|  | 63 | 51 ± 2 | 51 ± 2 | 52 ± 2 | 51 ± 2 | 49 ± 2 | 53 ± 2 | 53 ± 3 | 53 ± 3 | 53 ± 3 | 53 ± 3 | 53 ± 3 | NS |
|  | 94.5 | 55 ± 2 | 54 ± 2 | 54 ± 2 | 52 ± 2 | 53 ± 2 | 52 ± 2 | 50 ± 2 | 50 ± 3 | 51 ± 2 | 51 ± 3 | 50 ± 2 | NS |
| Gln | 0 | 624 ± 18 | 621 ± 28 | 614 ± 27 | 600 ± 27 | 602 ± 31 | 588 ± 31 | 592 ± 27 | 552 ± 40 | 582 ± 33 | 620 ± 25 | 630 ± 14 | NS |
|  | 63 | 666 ± 39 | 700 ± 34 | 707 ± 25 | 710 ± 28 | 712 ± 34 | 691 ± 24 | 703 ± 24 | 668 ± 32 | 667 ± 16 | 688 ± 16 | 685 ± 15 | NS |
|  | 94.5 | 653 ± 21 | 668 ± 25 | 677 ± 28 | 687 ± 27 | 696 ± 16 | 663 ± 15 | 666 ± 19 | 653 ± 24 | 671 ± 23 | 658 ± 10 | 685 ± 11 | NS |
| Glu | 0 | 26 ± 3 | 27 ± 6 | 33 ± 5 | 35 ± 4 | 40 ± 7 | 38 ± 7 | 38 ± 5 | 41 ± 6 | 42 ± 6 | 34 ± 4 | 34 ± 4 | NS |
|  | 63 | 24 ± 3 | 23 ± 2 | 27 ± 1 | 28 ± 3 | 25 ± 2 | 29 ± 4 | 30 ± 3 | 34 ± 3 | 35 ± 3 | 29 ± 3 | 30 ± 2 | NS |
|  | 94.5 | 30 ± 5 | 29 ± 7 | 31 ± 6 | 27 ± 5 | 28 ± 3 | 30 ± 3 | 31 ± 2 | 32 ± 3 | 33 ± 3 | 30 ± 2 | 28 ± 3 | NS |
| Gyl | 0 | 266 ± 7 | 266 ± 9 | 264 ± 5 | 259 ± 7 | 258 ± 8 | 254 ± 7 | 257 ± 9 | 242 ± 9 | 252 ± 12 | 255 ± 7 | 255 ± 4 | NS |
|  | 63 | 261 ± 6 | 273 ± 10 | 263 ± 11 | 255 ± 10 | 248 ± 10 | 249 ± 12 | 254 ± 14 | 246 ± 10 | 246 ± 11 | 252 ± 14 | 242 ± 16 | NS |
|  | 94.5 | 255 ± 4 | 253 ± 6 | 239 ± 6 | 233 ± 7 | 231 ± 5 | 222 ± 5 | 223 ± 6 | 221 ± 7 | 224 ± 6 | 219 ± 6 | 222 ± 6 | <0.05 |
| His | 0 | 90 ± 3 | 88 ± 4 | 88 ± 2 | 85 ± 3 | 86 ± 3 | 85 ± 3 | 87 ± 3 | 82 ± 4 | 85 ± 4 | 88 ± 3 | 88 ± 1 | NS |
|  | 63 | 94 ± 3 | 100 ± 4 | 100 ± 3 | 100 ± 3 | 94 ± 1 | 96 ± 3 | 96 ± 3 | 94 ± 3 | 94 ± 3 | 97 ± 4 | 95 ± 4 | NS |
|  | 94.5 | 98 ± 3 | 101 ± 3 | 100 ± 4 | 98 ± 4 | 95 ± 2 | 91 ± 3 | 90 ± 2 | 90 ± 3 | 93 ± 4 | 90 ± 2 | 91 ± 4 | NS |
| Lys | 0 | 220 ± 8 | 219 ± 11 | 216 ± 10 | 208 ± 10 | 208 ± 11 | 204 ± 11 | 207 ± 9 | 196 ± 11 | 202 ± 10 | 204 ± 9 | 206 ± 6 | NS |
|  | 63 | 235 ± 5 | 251 ± 6 | 251 ± 5 | 248 ± 5 | 241 ± 3 | 241 ± 6 | 247 ± 7 | 232 ± 8 | 232 ± 7 | 227 ± 9 | 216 ± 10 | <0.05 |
|  | 94.5 | 236 ± 6 | 242 ± 8 | 244 ± 9 | 245 ± 10 | 245 ± 7 | 233 ± 8 | 229 ± 8 | 223 ± 10 | 224 ± 10 | 205 ± 9 | 201 ± 6 | <0.05 |
| Pro | 0 | 147 ± 8 | 147 ± 8 | 144 ± 5 | 145 ± 7 | 141 ± 7 | 142 ± 7 | 145 ± 9 | 137 ± 9 | 137 ± 8 | 134 ± 7 | 130 ± 6 | NS |
|  | 63 | 173 ± 11 | 180 ± 10 | 170 ± 11 | 168 ± 10 | 161 ± 11 | 158 ± 10 | 159 ± 10 | 153 ± 13 | 150 ± 11 | 148 ± 9 | 142 ± 11 | NS |
|  | 94.5 | 165 ± 7 | 165 ± 10 | 157 ± 10 | 152 ± 10 | 147 ± 8 | 142 ± 8 | 141 ± 9 | 137 ± 10 | 138 ± 9 | 131 ± 9 | 132 ± 9 | NS |
| Ser | 0 | 129 ± 6 | 129 ± 6 | 129 ± 6 | 126 ± 6 | 125 ± 5 | 124 ± 5 | 126 ± 7 | 121 ± 6 | 125 ± 7 | 125 ± 5 | 126 ± 5 | NS |
|  | 63 | 121 ± 7 | 125 ± 9 | 124 ± 9 | 121 ± 9 | 116 ± 8 | 117 ± 9 | 117 ± 10 | 113 ± 7 | 113 ± 9 | 112 ± 8 | 112 ± 10 | NS |
|  | 94.5 | 121 ± 7 | 122 ± 8 | 118 ± 8 | 115 ± 8 | 111 ± 6 | 107 ± 5 | 109 ± 9 | 108 ± 5 | 107 ± 6 | 104 ± 4 | 104 ± 5 | NS |
| Thr | 0 | 160 ± 8 | 159 ± 10 | 157 ± 9 | 151 ± 9 | 150 ± 9 | 147 ± 10 | 149 ± 9 | 142 ± 10 | 146 ± 10 | 149 ± 9 | 149 ± 7 | NS |
|  | 63 | 150 ± 3 | 157 ± 4 | 153 ± 5 | 147 ± 4 | 142 ± 5 | 140 ± 4 | 141 ± 4 | 134 ± 6 | 133 ± 6 | 134 ± 5 | 130 ± 7 | NS |
|  | 94.5 | 151 ± 6 | 151 ± 6 | 145 ± 6 | 141 ± 5 | 138 ± 5 | 130 ± 6 | 129 ± 5 | 126 ± 7 | 127 ± 7 | 123 ± 6 | 123 ± 4 | <0.05 |

Values are means ± SE. *P* values were calculated for changes over time within each dose of leucine ingestion by one-way rmANOVA. NS stands for not significant.
