# Supplementary material for: Bolus ingestion of individual branched-chain amino acids alters plasma amino acid profiles in young healthy men
Source: Springerplus. 2014 Jan 17;3:35. doi: 10.1186/2193-1801-3-35 (PMC4320164; doi:10.1186/2193-1801-3-35)
Supplement: Supplementary file 2 — Additional file 2: Online Resource 1. Concentrations of plasma components measured other than amino acids in the leucine ingestion experiments. Online Resource 2. Concentrations of plasma components measured other than amino acids in the isoleucine ingestion experiments. Online Resource 3. Concentrations of plasma components measured other than amino acids in the valine ingestion experiments. Online Resource 4. Concentrations of plasma components measured other than amino acids in the mixed BCAA ingestion experiments. (DOCX 357 KB) [file 40064_2013_1453_MOESM2_ESM.docx]

Article title: **Bolus ingestion of individual branched-chain amino acids alters plasma amino acid profiles in young healthy men**

Journal name: **SplingerPlus**

Authors: Takuya Matsumoto^1^, Koichi Nakamura^2^, Hideki Matsumoto^3^, Ryosei Sakai^3^, Tomomi Kuwahara^3^, Yoshihiro Kadota^2^, Yasuyuki Kitaura^2^, Juichi Sato^1^, and Yoshiharu Shimomura^2^*

Affiliation: ^1^ Department of General Medicine/Family and Community Medicine, Nagoya University Graduate School of Medicine, Nagoya 466-8560, Japan; ^2^ Laboratory of Nutritional Biochemistry, Department of Applied Molecular Biosciences, Graduate School of Bioagricultural Sciences, Nagoya University, Nagoya 464-8601, Japan; and ^3^ Institute for Innovation, Ajinomoto Co., Inc., Kawasaki 210-8681, Japan

*To whom correspondence should be addressed: E-mail: [shimo@agr.nagoya-u.ac.jp](mailto:shimo@agr.nagoya-u.ac.jp)
